# Simple rules for evidence translation in complex systems: A qualitative study

**DOI:** 10.1186/s12916-018-1076-9

**Published:** 2018-06-20

**Authors:** Julie E. Reed, Cathy Howe, Cathal Doyle, Derek Bell

**Affiliations:** 1 National Institute of Health Research (NIHR) Collaboration for Leadership in Applied Health Research and Care (CLAHRC) Northwest London, Chelsea, London, UK; 2 Westminster Hospital, Imperial College, London, SW10 9NH UK

**Keywords:** Complex systems, Complexity theory, Complex adaptive systems, Framework, Evidence translation, Implementation, Quality improvement

## Abstract

**Background:**

Ensuring patients benefit from the latest medical and technical advances remains a major challenge, with rational-linear and reductionist approaches to translating evidence into practice proving inefficient and ineffective. Complexity thinking, which emphasises interconnectedness and unpredictability, offers insights to inform evidence translation theories and strategies. Drawing on detailed insights into complex micro-systems, this research aimed to advance empirical and theoretical understanding of the reality of making and sustaining improvements in complex healthcare systems.

**Methods:**

Using analytical auto-ethnography, including documentary analysis and literature review, we assimilated learning from 5 years of observation of 22 evidence translation projects (UK). We used a grounded theory approach to develop substantive theory and a conceptual framework. Results were interpreted using complexity theory and ‘simple rules’ were identified reflecting the practical strategies that enhanced project progress.

**Results:**

The framework for Successful Healthcare Improvement From Translating Evidence in complex systems (SHIFT-Evidence) positions the challenge of evidence translation within the dynamic context of the health system. SHIFT-Evidence is summarised by three strategic principles, namely (1) ‘act scientifically and pragmatically’ – knowledge of existing evidence needs to be combined with knowledge of the unique initial conditions of a system, and interventions need to adapt as the complex system responds and learning emerges about unpredictable effects; (2) ‘embrace complexity’ – evidence-based interventions only work if related practices and processes of care within the complex system are functional, and evidence-translation efforts need to identify and address any problems with usual care, recognising that this typically includes a range of interdependent parts of the system; and (3) ‘engage and empower’ – evidence translation and system navigation requires commitment and insights from staff and patients with experience of the local system, and changes need to align with their motivations and concerns. Twelve associated ‘simple rules’ are presented to provide actionable guidance to support evidence translation and improvement in complex systems.

**Conclusion:**

By recognising how agency, interconnectedness and unpredictability influences evidence translation in complex systems, SHIFT-Evidence provides a tool to guide practice and research. The ‘simple rules’ have potential to provide a common platform for academics, practitioners, patients and policymakers to collaborate when intervening to achieve improvements in healthcare.

**Electronic supplementary material:**

The online version of this article (10.1186/s12916-018-1076-9) contains supplementary material, which is available to authorized users.

## Background

There is an urgent need to improve the delivery of high quality healthcare, including the need to improve patient safety and reduce harm [1–3], to ensure care is patient centred and compassionate [4, 5], to improve health and wellbeing [6], and to reduce inequalities at the local, regional, national and global scale [7–9], all within an increasingly constrained financial environment [10, 11].

To address these challenges, there is a need to bridge the gap between the production of research evidence and the consistent delivery of evidence-based care in routine practice [12–15]. There is growing acknowledgement that translation of evidence is often ineffective and inefficient, and there is a need to develop a scientific and practical understanding of how to implement evidence into practice and achieve fast and reliable improvements in care [16–18].

Traditional approaches to translating evidence into practice have taken a rational-linear approach (where knowledge is created by one set of experts and passed on to another set to be implemented) [19, 20]. Evaluations have focused on identifying simple causal relationships between interventions and outcomes, aiming to produce generalisable knowledge about what works [16]. To establish causal relationships, studies tend to be conducted in controlled environments, where interference from context variables is considered problematic and controlled for by randomisation and protocol design [17].

It is increasingly recognised that context matters; having an ‘appropriate’ context can support an intervention achieve its outcome [21]. Approaches to translating evidence into practice have taken an interest in how interventions can be adapted to work in different settings [22, 23], and many researchers have turned to realist evaluations in an attempt to understand ‘what works, for whom, in what settings’ and establish more nuanced and caveated causal statements [21, 24].

When designing intervention and implementation strategies, as well as when conducting rigorous evaluations, there is a tendency to reduce messy real world situations into the individual component parts in an attempt to determine the relationships between them. Doing so risks overlooking the complex and intricate patterns that emerge from their interactions.

Complexity sciences provide an alternative approach to studying interventions in complex systems such as healthcare. Complexity science originated in physical chemistry as a ‘push-back’ against traditional reductionist approaches [25]. Put simply, life is more than molecules and atoms – it is the complex patterns of organisation that emerge between them [26, 27]. Similarly, it has been proposed that healthcare can be considered as a complex system [28, 29] (or complex adaptive system) [30, 31], with the whole being more than simply the sum of its parts. On their own, the professionals, equipment and devices in any healthcare setting achieve nothing; it is the interactions between them, and with patients, that result in the delivery of care.

Complex systems are characterised as a dynamic network of agents acting in parallel, constantly reacting to what the other agents are doing, which in turn influences the behaviour of the network as a whole [32]. The interconnected nature of their interactions can lead to uncertainty and surprise as systems self-organise and evolve over time in response to internal and external stimuli and feedback loops [28, 33]. This non-linearity means that complex systems can defy orchestrated intervention, wherein seemingly obvious solutions can have minimal impact on system behaviour (e.g. policy resistance) [34], whilst small changes can have big unanticipated consequences. Such systems have strong historical path dependencies, meaning that initial conditions are influenced by historic events and patterns, and that they can markedly influence what happens in the future.

On the one hand, complex systems are highly dynamic, continually responding and adapting to internal and external stimuli. While, on the other, they can demonstrate inertia where embedded behaviours remain unchanged and even temporary perturbations or major structural alterations can fail to disrupt existing norms [34, 35]. From these unpredictable and evolving systems emerge patterns, behaviours, structures and routines which define the system and guide behaviours within it [33, 36]. Complexity theorists propose that ‘simple rules’ offer a means of understanding and managaing the emergent behaviour of complex systems [26, 34].

The use of complexity science as a lens to understand healthcare systems is increasing [36]. To date, research studies have predominantly focused on describing healthcare systems as complex, yet there is less understanding of how to predict or intervene [37]. Advances have tended to be theoretical with the purpose of guiding evaluations or further research [38, 39]. Whilst there is an increased use of the term complexity, there is little evidence that the concepts of complex systems have been applied to the design of interventions or implementation strategies [40]. As such, Braithwaite et al. [36] have called for a greater clarity about how to study and apply the principles of complex systems in practice.

This study aims to develop a deeper explanation of evidence translation in healthcare using a complex systems lens, thereby contributing to both the fields of implementation science and complexity science. Drawing on detailed insights into complex micro-systems, this research advances empirical and theoretical understanding. A primary focus is given to understanding the implications of complexity theory with an objective of identifying a series of ‘simple rules’ about how to intervene in complex systems. The ‘simple rules’ aim to make complexity navigable (whilst recognising that it will never be simple), providing actionable guidance to both practice and research.

## Methods

### Study design

**Fig. 1:**
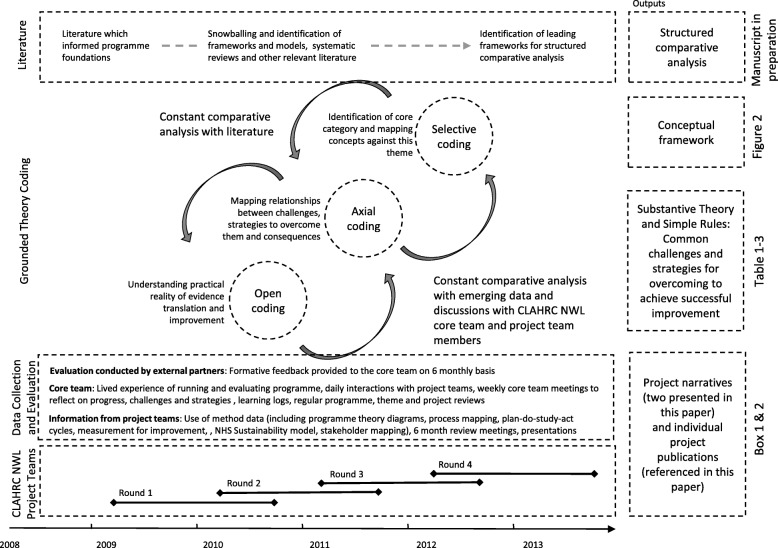
A schematic representation of data collection and coding approach

The study was conducted using an analytical auto-ethnography and grounded theory approach (Fig. 1). An analytical auto-ethnographic approach was adopted reflecting that the authors of this paper were full members of the research setting (conducting ethnography of ‘our own people’ as members of the "core team" (Fig. 1)), visible as such a member in published texts, and committed to developing theoretical understandings of broader social phenomena [41].

Empirical data was collected through participant observation and document analysis of the National Institute of Health Research (NIHR) Collaboration for Leadership in Applied Health Research and Care (CLAHRC), Northwest London (NWL) programme (UK), and 22 evidence translation projects (Additional file 1). This allowed direct access to and observation of actions, events, scenes and people in real-time over a 5-year period, with opportunities to follow-up on emergent patterns and problems. Concurrently, extensive literature was reviewed using a snowballing approach to identify frameworks, models, systematic reviews and other relevant literature (further details on data collection and literature review can be found in Additional file 2).

A grounded theory approach guided the data collection and analysis [42, 43]. Data was analysed using open, axial and selective coding, in parallel with theoretical sampling, to explore emergent categories and themes over time. This iterative analysis led to a process of ‘abduction’ to make sense of material that did not ‘fit’ into pre-established categories (including published frameworks and theories), thereby reconceptualising the challenge of evidence translation and improvement into a new substantive theory (to provide explanations and predictions related to the specific context of study) and conceptual framework (indicating how aspects of the theory are connected to each other). Further details are provided in Additional files 2 and 3.

This exploratory research approach was chosen to ensure that the resulting findings were empirically informed and theoretically grounded in the practical reality of evidence translation and improvement in real world (complex) settings. We chose not to build exclusively on any existing theories as no single existing framework fit well with our experiences. Whilst several fields of study were relevant, no single frameworks brought together concepts from different fields, including knowledge translation, implementation, improvement and complexity.

Results from the grounded theory analysis were interpreted through complex systems thinking [26, 28, 34, 35]. Emphasis was placed on developing a series of ‘simple rules’, which were identified through establishing relationships between challenges experienced by the project teams, and the actions and strategies that, if taken, had a positive effect on project progress and outcomes or, if they were absent or overlooked, were observed to have a detrimental impact.

### Setting

The NIHR established the CLAHRC programme in England to accelerate the translation of evidence into practice for the benefit of patients. Thirteen regional CLAHRC programmes were funded, each led by academic and healthcare partnerships and with autonomy to decide how they would approach ‘closing’ the translational gap [44–46].

The CLAHRC NWL approach brought together healthcare staff, including clinical, managerial and support staff (hereafter referred to as ‘staff’) with patients, carers, family members and the wider community (hereafter, ‘patients’) and academic partners from a diverse range of disciplines (hereafter, ‘academics’) into project teams of 5–15 people to translate evidence into practice in their local micro-systems. Project teams used a suite of quality improvement tools and methods, including the model for improvement, action-effect diagrams and plan-do-study-act cycles, process mapping, statistical process control, stakeholder engagement, and patient and public involvement combined with iterative evaluation, to guide and support the implementation process [47–51].

During the first 5 years of the CLAHRC NWL (2008–2013), 22 diverse topics considered of clinical importance were explored with 55 teams over four rounds of 18-month projects (Fig. 1) in various settings (acute, community, primary care, mental health, etc.) (Additional file 1). All projects had a common goal of translating existing evidence into practice to achieve improvements in quality of care provision, with the aspiration of delivering corresponding improvements in patient outcomes. Two detailed case study examples are presented in the results section (Boxes 1 and 2).

This paper represents a consolidation of cross-project learning from the programme and peer-reviewed literature. Existing publications relating to evaluation of individual projects, cross-project analysis, use of quality improvement approaches, and external programme evaluations are listed in Additional file 1.

## Results

Results are divided into two sections. Firstly, the new conceptual framework Successful Healthcare Improvements From Translation of Evidence into practice (SHIFT-Evidence) is presented, introducing the three strategic principles of the framework, namely ‘act scientifically and pragmatically’, ‘embrace complexity’ and ‘engage and empower’, and the 12 ‘simple rules’.

Secondly, there is a detailed presentation of the 12 ‘simple rules’ and accompanying substantive theory. The results demonstrate how the theory and rules emerged from the empirical data and how understanding is enhanced by application of a complex systems lens. The presentation of the rules and substantive theory is accompanied by two illustrative case examples from CLAHRC NWL projects to bring to life the practical reality of evidence translation.

### A conceptual framework for SHIFT-Evidence

The theory of SHIFT-Evidence can be summarised as follows: to achieve successful improvements from evidence translation in healthcare, it is necessary to ‘act scientifically and pragmatically’ whilst ‘embracing the complexity’ of the setting in which change takes place and ‘engaging and empowering’ those responsible for and affected by the change.

SHIFT-Evidence reflects the nature of work and breadth of effort required to translate evidence into complex systems. The findings revealed that attention and effort was often drawn away from the original project focus in directions that were not anticipated in advance, such as dependent issues relating to people, processes or structures, or to resolve existing problems with ‘usual care’. We established that failure to resolve these issues compromised the success of an intervention and diminished the ability to draw useful conclusions about the effectiveness of any intervention in a real-world setting. As such, the SHIFT-Evidence framework is conceptually based on the premise that the implementation of evidence-based interventions is not necessarily sufficient to achieve improvements in care, and that it is not possible to fully anticipate what changes will be required in any individual setting. In short, evidence translation and wider systems improvement are inextricably linked within complex systems.

The accumulating data about the ‘daily realities’ of evidence translation and improvement required us to reconceptualise our understanding of the problem, and associated potential solutions. Our focus moved from evidence-based medicine and interventions, to focusing on the complexity of the systems within which we hoped to intervene. As such, literature relating to complex systems thinking grew in importance over time to become the primary lens by which we were able to make sense of our experiences (further details on this process of reconceptualisation are provided in Additional file 2).

Reflecting this reconceptualisation, ‘act scientifically and pragmatically’ was identified as the core category for selective coding. It was chosen to reflect the interaction between our starting world view (the need to use scientific evidence) and our core learning (the need to understand and respond to the constraints and opportunities of the local system). Our analysis indicated the tension between these perspectives, and also the opportunity for increased synergy between them, as follows:

- An underlying tension was observed in the literature and in our empirical data between the production and use of generalisable knowledge (influenced by positivist and realist philosophical perspectives) and local context-specific problem solving (influenced by pragmatist and participatory philosophical perspectives).
- We recognised the value of drawing insights from both perspectives. Effective improvement initiatives can benefit from drawing on a scientific knowledge base (evidence-based medicine, or other knowledge of effective interventions or change processes), and from making pragmatic adjustments in line with the opportunities and constraints of the actual setting for the change.
- The change process can be guided by applying aspects of the scientific method at a local level so that clear aims and measures guide structured experimental processes to assess, learn and inform next steps. This resonates with the pragmatist notion of science to solve local problems of societal importance [52], and with the complexity literature notion of the *“science of muddling through*” in dynamic and evolving systems [53].

Two further important key categories were identified, namely ‘embrace complexity’ and ‘engage and empower’. These three high level conceptual categories are termed strategic principles, reflecting the guidance on how to conduct and research evidence translation and improvement in complex systems. These principles are underpinned by 12 associated ‘simple rules’, which describe the actions required to achieve each strategic principle.

The three strategic principles and 12 ‘simple rules’ are the following:

**Act scientifically and pragmatically:** Knowledge of existing evidence needs to be combined with knowledge of the unique initial conditions of a system. Interventions need to adapt as the complex system responds and learning emerges about unpredictable effects. This strategic principle reflects the high level stages of an improvement initiative through the four simple rules:

- Understand problems and opportunities
- Identify, test and iteratively develop potential solutions
- Assess whether improvement is achieved, and capture and share learning
- Invest in continual improvement

**Embrace complexity:** Evidence-based interventions only work if related practices and processes of care within the complex system are functional. Evidence-translation efforts need to identify and address existing problems with usual care, recognising that this typically includes a range of interdependent parts of the system. This principle emphasises the need to investigate and understand the uniqueness of each local system and respond to complexity from the micro- to macro-system as reflected by the four rules:

- Understand processes and practices of care
- Understand the types and sources of variation
- Identify systemic issues
- Seek political, strategic and financial alignment

**Engage and empower:** Evidence translation and system navigation requires commitment and insights from staff and patients with experience of the local system. Changes need to align with their motivations and concerns. The four rules reflect factors that influence engagement at an individual and team level through to supporting infrastructure and organisational level:

- Actively engage those responsible for and affected by change
- Facilitate dialogue
- Foster a culture of willingness to learn and freedom to act
- Provide headroom, resources, training and support

**Fig. 2:**
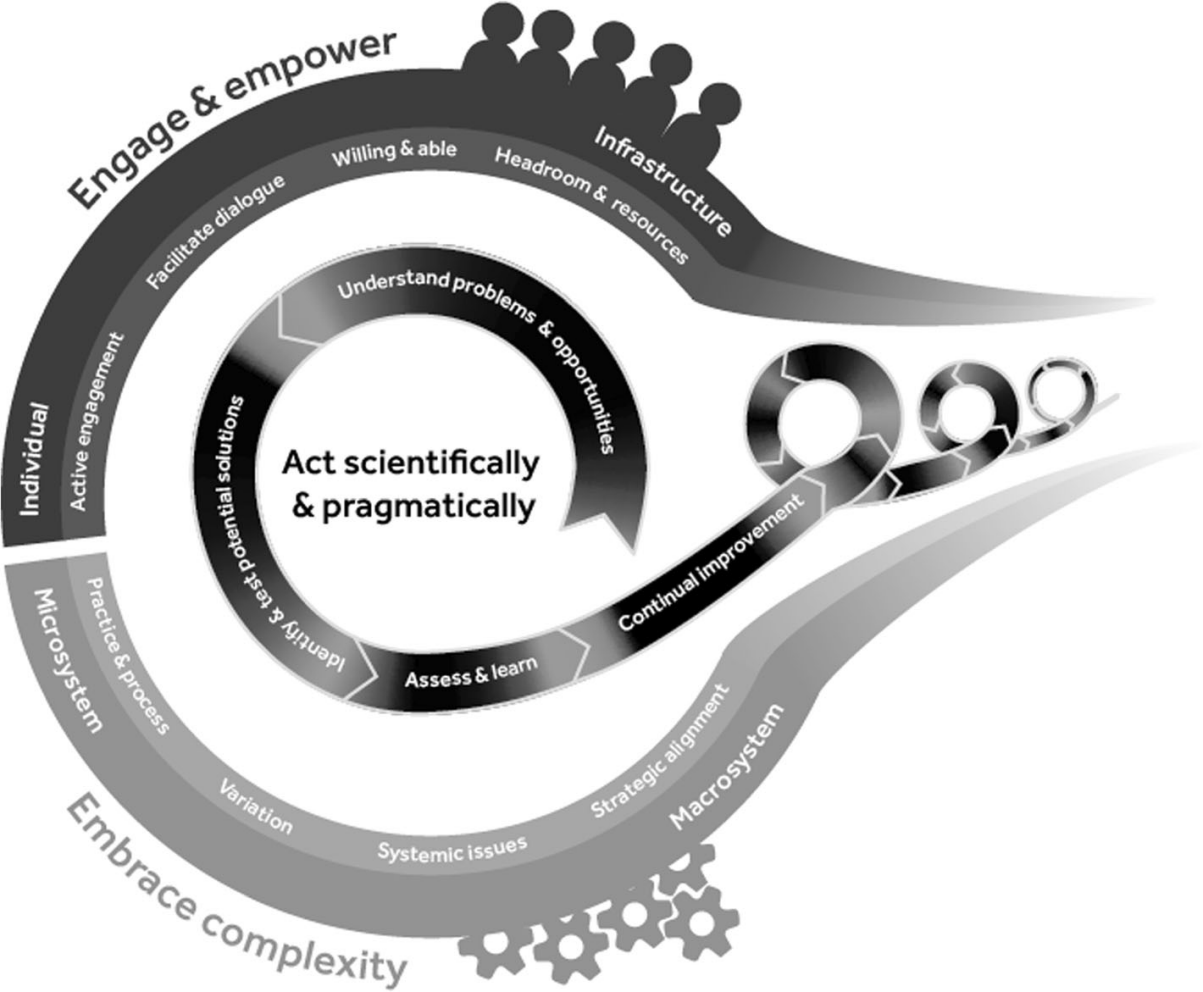
A schematic representing the SHIFT-Evidence conceptual framework including the three strategic principles (act scientifically and pragmatically, embrace complexity, and engage and empower) with the 12 associated ‘simple rules’. The diagram represents the continual iterative process of evidence translation and improvement in healthcare

**Relationship between SHIFT-Evidence principles:** The process of evidence translation and improvement, as represented in SHIFT-Evidence, is intended to be a progressive iterative process. The ‘simple rules’ provide a conceptual framework to guide practice and research in complex systems, responding to emergent challenges and capturing generative learning (Fig. 2). In practice, feedback loops exist between each of the rules as learning emerges about the changes required and effectiveness of interventions. Few improvement initiatives follow a smooth linear pattern.

Our hypothesis is that all SHIFT-Evidence strategic principles and ‘simple rules’ are necessary to achieve successful and sustained improvements in care, and are interdependent. For example, ‘active engagement’ of healthcare professionals and patients is necessary to fully ‘understand practices and processes of care’. Equally, ‘active engagement’ of staff may reveal that their priorities do not align with current ‘strategic, political and financial’ incentives, and vice versa. Our hypothesis implies that such tensions, if unresolved, will negatively impact success.

### Project narratives, common challenges and simple rules

Two of the 22 CLAHRC NWL project narratives are presented as detailed examples to illustrate the practical reality of evidence translation and improvement (Box 1 and 2). Both demonstrated measurable success against their original objectives, although each encountered unexpected obstacles. This is followed by presentation of the 12 simple rules, describing how the simple rules relate to the project narratives and substantive theory (Tables 1, 2 and 3), and reflecting on insights provided by complex systems thinking.

**Table 1:** Substantive theory for acting scientifically and pragmatically – challenges and corresponding actions required for successful evidence translation and improvement

Act scientifically and pragmatically	
Common challenges	Simple rules: strategies for overcoming challenges
Pre-selected interventions may not solve the problems of the local system - People will not be motivated to change if they do not perceive a problem exists, or if wider concerns prevail - Varying perceptions of current practice - Conflicting views of problem and improvement approach	Understand the problem and opportunities - Draw on evidence and local knowledge to understand the problem and opportunities - Understand perceptions of local needs and priorities - Identify common improvement goals
‘Evidence’ and interventions need to be perceived as locally relevant and actionable - Varying perceptions of evidence - Interventions may not be used or work effectively - Interventions need to fit with or modify existing practices, behaviours and competencies - Interconnected challenges emerge as changes are made - Changes have unintended consequences - Multiple interventions are likely to be required	Identify, test and iteratively develop potential solutions - Identify intervention ideas based on evidence and build theory of change - Incremental experimental approach to introduce changes - Identify and respond to emergent challenges - Identify adverse effects in other parts of the system - Modify and refine change theory in response to learning - Review and balance investment of effort across problems and potential interventions to maximise impact
Individual perceptions of system performance are unreliable - System performance and characteristics can be hard to see from any individual perspective as they do not take into account system complexity - Objective measures can reveal how the system is performing but may not reveal why or what changes are required - A lack of data and narrative limits learning, including within teams, organisations or for research	Assess whether improvement is achieved, capture and share learning - Carefully select a small number of measures as an objective indication of system performance - Use regular measurement to assess impact and inform actions - Use formal and informal methods to obtain feedback that can explain performance and guide future actions - Capture change narrative and use for organisational memory, to spread learning and to inform research
Interventions need to be reviewed and adapted as systems evolve over time - Healthcare is a continually changing dynamic system - Competing factors threaten long-term success - New evidence, priorities and opportunities emerge	Invest in continual improvement - Anticipate, plan and monitor for threats to sustainability - Proactively identify and incorporate new evidence - Continually respond to new ‘problems’ and opportunities

**Table 2:** Substantive theory for embracing complexity – challenges and corresponding actions required for successful evidence translation and improvement

Embrace complexity	
Common challenges	Simple rules: strategies for overcoming challenges
Interventions do not work on their own – they need to fit with practices and processes of care - Interventions need to be used by people and are dependent on other processes and practices - Interventions interact with complex processes and established practices of professions and organisations	Understand practices and processes of care - Understand what is actually happening and identify interdependent practices and processes - Consider fit of new or modified interventions and use to inform intervention design, implementation activities, and ongoing learning and actions to drive improvement
There is rarely a single, standardised, way by which care is delivered - Agents within the system are constantly interacting and responding to each other and to internal and external stimuli - This results in inherent levels of variation within healthcare systems, even when standard processes exist - People have to make decisions and take actions in real world (imperfect) conditions	Understand types and sources of variation - Natural variation needs to be understood to inform intervention design and implementation - Identify what variation is (un)acceptable and what improvements are required - Use data, observations and feedback on variations to learn and assess whether progress is being made
It cannot be assumed that dependent processes or systems are working well - To achieve the original improvement goal other problems or related issues may need to take priority - Practices and processes are often sub-optimal and may require improvement to support evidence implementation - Systemic problems can be hard to overcome and may challenge assumptions or current practices and cultures - Not all systemic problems can be addressed	Identify systemic issues - Be vigilant for systemic issues as learning emerges - Consider what is within the project team’s sphere of influence, and where additional support is needed - Use learning to influence planning and design, and where necessary how to function within system constraints
Any intervention will compete for attention and resources with other initiatives or requirements - Attention and resources are limited and initiatives need to work within system constraints - Initiatives will always ‘compete’ with other priorities and may fail without appropriate support and backing - Managerial, financial, strategic and political decisions and motives may work in support or against an initiative	Seek political, strategic and financial alignment - Recognise system constraints and be realistic about what can be achieved given finite resource and competing priorities - Consider where improvement might have greatest impact - Understand negative or positive impacts of political, strategic or financial incentives on behaviours and use this to inform the design of interventions - Where possible, seek alignment and consider how to secure support and continued investment

**Table 3:** Substantive theory for engaging and empowering – challenges and corresponding actions required for successful evidence translation and improvement

Engage and empower	
Common challenges	Simple rules: strategies for overcoming challenges
If people are not motivated, change will not take place, and without their engagement, insights will be lost - People will resist changes that do not fit with their perceptions of what change is required and it is necessary to understand their emotional responses - Healthcare professionals and patients hold local knowledge about how care practices and processes work - No one person can ‘see’ the whole system, but each person can provide valuable insights	Actively engage those responsible for and affected by change - Understand what really matters to people and connect with emotional drivers for change - Engage people in identifying and understanding problems so they ‘own’ the rationale for change, to harness their knowledge and input to design and test solutions and gather feedback on what does or does not work
Expect conflict and tension - Sharing knowledge and making sense of different, conflicting, perspectives is challenging; different professional groups have different norms and languages - Power differentials exist and it takes time to build trust and relationships to support meaningful dialogue - Patient’s experiential knowledge is not always valued	Facilitate dialogue - Create a safe environment for people to share their views and increase common understanding of the system - Promoting listening and constructive dialogue and building trust, relationships and partnership - Facilitate discussion and engage people in active reflection and discussion at all stages of design, conduct and evaluation, and respect emotionally charged responses
Underlying expectations are to get it right, first time, quickly - Focus on centralised leadership, command and control - Expectation of ‘positive results’ and importance placed on ‘being right’, with a lack of constructive challenge - Limited opportunities to share opinions or concerns, and lack of permission or support to try their own ideas - Judgemental cultures can represses learning	Build a culture of willingness to learn and freedom to act - Provide healthcare professionals and patients with the freedom and support to investigate and take action - Support the development of a learning culture which is willing and able to conduct improvement work - Encourage learning from failure as well as success and openness about real performance and problems
Improving complex systems takes time, effort and reflection - Care professionals rarely have time to consider how different parts of the system work together beyond their professional group, unit or department - Change takes time, and requires different skill sets to those in which professionals are traditionally trained - Competently enacting new practices requires training and practice - Organisations lack improvement infrastructure	Provide headroom, resources, training and support - Provide professionals with time out from daily practice spaces to collaborate with each other and patients - Provide training and support for new interventions or changes to practice to build competence and confidence - Build skills and competencies for improvement in individuals and at an organisational level, including specialist skills and data infrastructure

The project narrative in Box 1 outlines the challenges to embedding evidence-based practices and achieving improvements in care quality for patients with community-acquired pneumonia (CAP).

The second project (Box 2) illustrates the complexity of healthcare systems and how this was experienced by a clinical team attempting to improve medicines management (MM) for patients following discharge from hospital.

### Act scientifically and pragmatically

The strategic principle ‘act scientifically and pragmatically’ demonstrates that knowledge of existing evidence is only one part of the effort required to achieve sustainable improvements in care in complex systems.

#### Understand the problem and opportunities

The two case studies reveal the challenges of introducing evidence-based practices or interventions into complex systems, and demonstrate how any intervention is sensitive to the unique initial conditions of the local system.

The MM project narrative shows how the interconnectivity of different system elements influenced the work that was required to improve the system; the desired intervention (a follow-up phone call) could not be initiated until dependent processes (medicines reconciliation at discharge from hospital) were improved.

The CAP project narrative demonstrates how the autonomy of individual agents working in the system challenged the introduction of the care bundle intervention; at the outset of the project, there was little incentive or motivation to take action to address a problem many perceived did not exist. Baseline data was required to help create tension for change by demonstrating the extent of the local problem.

Linear models for the spread and scale-up of evidence-based practices assume the same intervention can be applied to the same problem in multiple settings. Understanding the consequences of working in complex systems challenges these assumptions; the historical origins and path-dependency of any given system means that somewhat different problems or configurations of problems and opportunities will exist in any given setting [35]. To gain traction, effort needs to be invested in understanding priority issues and areas for improvement within the local system, and any interventions need to be perceived as relevant and actionable by system agents [54].

#### Identify, test and iteratively develop potential solutions

Both project narratives reveal how system interconnectedness presented a challenge to fully anticipating which changes were required. This was reflected at two levels. Firstly, each intervention needed to be refined and adapted in response to emergent learning about local practice and to fit with established processes (e.g. modifications to the design of the CAP care bundle or MM reconciliation form). Secondly, each project needed to address multiple parallel or dependent issues beyond the original scope of their project to achieve their improvement goal (e.g. CAP project needed to address antibiotic prescribing policies and microbiological test ordering processes, MM project needed to address pharmacy staffing rotas and the roles and responsibilities of junior doctors).

Observing evidence-translation through a complexity lens therefore suggests the need to consider multiple strategies for intervening and the considerable effort that is required to support the uptake of any specific evidence-based practices. System understanding emerges over time, and often in unexpected ways, through testing intervention ideas in practice and responding to insights and challenges that are often difficult to anticipate, reflecting tacit knowledge or deep-seated routines and cultural practices [55].

#### Assess whether improvement is achieved, and capture and share learning

Both project narratives reveal the challenges of gauging performance in a complex system from an individual perspective. Objective measurement revealed in both instances that standards of care were lower than anticipated (CAP patients receiving evidence-based care standards; MM patients with fully reconciled medicines at discharge). These findings provided an insight into ‘hidden’ system performance, and reflects, despite the good intentions and hard work from individual agents, the challenges of coordinating collective behaviour of agents towards a common goal.

The need for measurement to guide improvement efforts also applied when sharing learning. As the CAP care bundle rolled out to local hospitals, the original site shared their experiences of developing the intervention and implementation. Whilst some learning was captured formally in versions of the care bundle form and summaries of the actions taken, the written material provided only a partial representation of the issues encountered and their resolution. Much of the learning about what had happened was shared through dialogue. Even armed with this learning, local sites essentially started from the beginning, understanding their own local problems and opportunities, building will and motivation to adopt new ways of working, and adapting intervention concepts to work in their local setting.

Given the uncertainty and unpredictability of intervening in complex systems, objective measures can provide a driving force to inform project progress. Rather than assuming that interventions were used and effective, measurement supported teams to accurately assess progress towards their goal and revise and adapt interventions and implementation approaches in light of results [56].

#### Invest in continual improvement

The challenge of sustaining initial improvements required teams to navigate both system inertia, attempting to pull practices back to ‘the way things have always been done’, and system evolution in response to internal and external stimuli.

Whilst all CAP sites achieved initial success, not all sites sustained those gains. High staff turnover was a persistent challenge to maintaining improvements with systems suffering ‘memory loss’, particularly when junior doctors leave en masse during clinical rotations. Other challenges included the consistency of clinical and managerial leadership, their ability to maintain a high profile for the work, and to cope when other emerging and often competing priorities drew attention to other parts of the system. Sites that did sustain were able to connect care bundle use to other substantive practices such as standardised admission processes and a history of care bundle use for other clinical presentations.

This learning demonstrates that improvements in care are not static; indeed, the complex and adaptive nature of healthcare systems means emergent events may threaten or enhance achievements [57]. Translation cannot be seen as a one-off activity and on-going monitoring and review needs to guide actions to adapt to system dynamics and support long-term success [58]. This learning is summarised in our substantive theory presented in Table 1.

### Embrace complexity

The strategic principle ‘embrace complexity’ demonstrates that evidence-based interventions only work if supporting or dependent practices and processes of care are working sufficiently well.

#### Understand practices and processes of care

The project narratives demonstrate that interventions do not exist in isolation, but need to fit with, and are dependent on, other practices and processes of care.

Initial perceptions of the project team leaders and other clinicians tended to view interventions in isolation from the system (MM perceived the follow-up phone call would be a standalone intervention to improve patient understanding of their medicines, and initial work of the CAP team focused exclusively on developing and perfecting details of the paper care bundle form). Once MM project team had identified the interdependency of the follow-up phone call with medicines reconciliation processes at discharge, they sought to understand why current practices were not working. They found that, although separate processes for documenting medicines reconciliation were routinely used with each individual staff group, they did not support communication and consolidation between staff groups. This was left to serendipity (e.g. being on the ward at the same time as another staff member) and personal effort to communicate and exchange information between professional groups. This insight led them to develop an additional intervention, namely a new shared form for medicines reconciliation that would be used by all four professional groups.

Complexity theories suggest that it is not possible to understand a system, or how to influence it, by reducing the system to its individual parts. As the projects progressed, it became increasingly apparent that project teams needed to look beyond individual competence or actions, to understand the complex interactions between individual agents, and the resulting patterns, that determine the quality of care [28].

#### Understand the types and sources of variation

A major challenge faced by the project teams was recognition that there is no single standardised way by which care is delivered. Whilst complex systems can give rise to regular patterns and ingrained behaviours, these are constantly perturbed by internal and external stimuli that systems adapt and respond to.

As the baseline data demonstrated, doctors’ knowledge of appropriate treatment for CAP patients did not translate into high quality care. The delivery of care needed to be reconceptualised as a series of hand-offs and interactions between multiple healthcare professionals (doctors, nurses, pharmacists, porters) each of which could be subject to various interruptions and delays whilst healthcare staff deal with multiple patients and competing priorities. Influencing factors ranged from the small acts of individual discretion (e.g. at what time a staff member took lunch break, how long they stopped to talk to a patient, or which order patients were seen in), to factors outside of any individuals immediate control (how many patients are admitted that day, the experience level of staff on shift, temporary staffing shortages (sickness, compassionate leave), chronic staffing shortages (funding, staff training and retention), and crisis events).

Investigation revealed that there were no routine processes for treating CAP. Each member of staff had developed individual approaches reflecting their personal knowledge of the system and relationships within it necessary to coordinate and deliver patient care. Introducing a shared standardised practice (the care bundle) helped to reduce variation but was not fail-safe and variation was still apparent, influenced by the factors listed above. The care bundle contributed to creating a more resilient process less likely to be affected by every day events such as interruptions or communication failures.

Intervening in complex systems requires an understanding of the variation inherent in all healthcare systems. Complex systems are dynamic and fluctuating, continually responding to internal and external stimuli, which means people have to make decisions and take action in real-world conditions. Rather than assuming standardised, idealised processes exist, it is necessary to understand and work with the complex reality of the settings in which care is delivered [59].

#### Identify systemic issues

The project narratives demonstrated that, even once interconnected and dependent processes and systems are identified, it cannot be assumed that they are working well.

The MM team discovered whole system issues with chains of dependencies, wherein phone calls depended on accurate information, accurate information depended on medicines reconciliation, and medicines reconciliation depended on staff coordination and joined-up procedures. Not all of these dependent, problematic, areas were within their direct control, and relationships had to be fostered with other key agents (e.g. educational leads, executive managers) to influence areas of concern. Some were considered unresolvable within the sphere of influence and timescales (e.g. interoperability of primary care and secondary care electronic health records) and were ‘parked’, or workarounds developed (e.g. where patients involved with the project developed a solution (patient-held medication records) that was not constrained by organisational or professional boundaries).

This demonstrates the nature of working in an open system. Not only is there interconnectedness within a system, but between various nested systems which connect and interact in a multitude of ways (e.g. the pharmacy system interacts with and is influenced by wider hospital systems, medical education systems, electronic record systems, etc.). Achieving an overall improvement required many other aspects of the system, and related systems, to be ‘fixed’. The original evidence-based intervention acted as a catalyst for a more comprehensive, complex and challenging system-wide analysis and an improvement process that required support and action from the wider organisation [60, 61].

#### Seek political, strategic and financial alignment

A persistent challenge faced by the project teams was that their individual areas of interest and interventions needed to compete for attention and resources with other initiatives or requirements.

Both projects were initially facilitated by financial support from the NIHR CLAHRC NWL programme, which created space and resource to test and develop interventions and capture an evidence base of their effectiveness. However, engaging already busy and fully committed clinical staff proved challenging, and given the system interdependencies, project teams needed to build strategic and political alignments with other system stakeholders to influence areas beyond their control.

The long-term sustainability of the projects was influenced by political, strategic and financial alignment. MM took advantage of changing political priorities to secure resources to support new ways of working and to increase awareness and perceptions of importance in frontline staff, and was able to sustain new medicines reconciliation practices. The sustainability of the CAP care bundle was variably influenced in the different organisations by their ability to align with key performance indicators, financial incentives or cost-saving initiatives.

Understanding complexity also means being aware of the constraints within the system. If more resources are consumed in one area, then another area will receive less. The finite amount of time, resource and attention within a system is already heavily committed with other wider organisational priorities, including managing service capacity to meet demand, achieving performance targets and responding to policy changes [62, 63], and implementation of multiple sources of evidence and innovations [64]. Evidence translation processes must consider organisational operating pressures and carefully reflect on where resources should be focused to achieve the maximum impact.

This learning is summarised in our substantive theory presented in Table 2.

### Engage and empower

The strategic principle ‘engage and empower’ demonstrates that evidence translation and system navigation requires commitment and insights from staff and patients with experience of the local care settings, and changes to a complex system need to align with their motivations and concerns.

#### Actively engage those responsible for and affected by change

Both projects experienced the harsh reality that if people are not motivated, change will not take place. They realised it was necessary to align with existing personal drivers or build motivation for change in order to get people to adopt new ways of working, and contribute insights and support to problem solving and overcoming obstacles.

In the CAP project, despite having motivated and embedded clinical leadership and support from a multi-disciplinary team, it was a challenge to engage other staff, and in particular other senior doctors. Doctors who believed they already knew how to treat CAP patients, were sceptical about the value of the intervention, and concerned that the care bundle was ‘dumbing down’ complex medical knowledge for junior doctors. Producing the care bundle intervention was not sufficient to instigate behaviour change, and it was rarely used. Engaging staff to understand and respond to their concerns, combined with regular use of measurement and feedback, supported on-going learning and generated local evidence to convince more sceptical individuals that the care bundle increased reliable delivery of evidence-based care. Investing time to engage staff was critical to the use of the intervention.

This example provides a powerful demonstration of the agency of individuals in a complex system. They are highly autonomous, skilled and opinionated individuals with significant discretion to choose what they do and how they do things. This enables them to evade new practices that they have not bought into (or do them in a tokenistic manner), whether initiated by colleagues or through top down directives.

Whilst engaging people can be challenging, the insights they provide are critical to understanding the problems and opportunities, evolving the intervention design and to identify dependent problems to address. In the MM project, patients provided insight into system problems that professionals had not been aware of. Much of the knowledge needed to understand why the problem existed, and how to overcome it, was held tacitly by frontline staff.

‘Seeing’ a complex system is hard. It is necessary to draw on local knowledge and practical wisdom to understand how different elements of care fit together, whilst recognising that each individual only experiences the aspects of a system with which they interact directly. No individual is capable of knowing all parts of a system.

Frontline staff and patients need to be central in the planning, design and conduct of evidence translation and quality improvement endeavours [65, 66]. People affected by change are those most invested in taking ownership and overcoming obstacles and barriers to ensure changes function at a local level [67, 68]. Identifying personal and emotional drivers, and aligning changes to those drivers, can ensure people remain motivated and persistent at times of challenge.

#### Facilitate dialogue

Bringing together different professional groups and patients may sound straightforward, but this was frequently experienced as challenging, and project teams learnt to anticipate conflict or tensions between different agents. For example, patients with CAP are routinely transferred within the critical first 4 h; therefore, treatment required coordination between the emergency department and the acute medical unit. Although staff from both departments were involved in the project, disputes emerged about who was responsible for initiating and completing the care bundle. Division of labour (partly driven by increasing specialisation) had exacerbated the boundaries between professions, units and organisations, each with their own beliefs, performance expectations and ‘territory’ to protect. Changes to established routines were perceived as threatening or distracting, or compromising professionals’ autonomy and ability to effectively perform their established roles. Dialogue between different ‘communities of practice’ and collaboration between professionals and patients often required facilitation [69, 70].

In complex systems, time is required to facilitate social sense-making, increasing understanding of each other’s perspectives and motives, and to learn how these can better coexist in the same system [71]. Whilst agents may frequently interact with one another, they rarely understand each other’s experiences of being in the system and the expectations, pressures and uncertainties they may face. Change affects individuals in different ways. Patients need to know how new care processes will affect them; staff need to understand how it can be incorporated into their current workload and how it will affect their status or professional identity [72].

#### Build a culture of willingness to learn and freedom to act

The teams we observed tended to work in high pressure environments with constrained resources and high performance standards and expectations. There were underlying expectations to get things right quickly the first time, which often repressed people’s ability to admit uncertainty or when things were not working well.

These behaviours were reflected in some project team members’ command and control leadership styles resulting from traditional hierarchies. Team members also tended to expect that change would be easy and quick. Many teams found it demoralising when their initial change ideas did not work straight away, or at the large number of barriers and obstacles that needed to be overcome in the process.

Successful teams tended to have the curiosity and persistence in the face of unexpected learning or set-backs. They also tended to be less hierarchical, where the views of all team members were listened to and valued and people were empowered to explore and solve problems. For example, the MM project discovered that, although individual professions were working hard, their collective endeavours failed to consistently deliver the high-quality care they valued. This was disappointing to the staff, but the team transformed this into energy for change. A culture focused on performance management may have repressed this discovery, denying the organisation an important opportunity to learn.

This reflects the inability to ‘control’ complex systems, or to reliably predict how to intervene to achieve a desired outcome. To be successful, it is necessary to have the humility to accept that the answers cannot be fully known in advance, to be willing to learn from experiments conducted within the local system, and to distribute leadership, engaging agents from across the system in the act of improving the system [73, 74].

#### Provide headroom, resources, training and support

Improving complex systems takes time, effort and reflection. Whilst healthcare professionals work to deliver care to the best of their ability within many constraints, they have little time to consider how the whole system functions. Many of the skills required to understand and intervene in complex systems (e.g. understanding processes and variation, team work) are not commonly taught to healthcare professionals or patients, and represent new ways of thinking that are often counter-cultural to prevailing norms [75].

These project narratives highlight that translation and improvement require space and time. Staff needed ‘headroom’ away from busy practice, time to think, to engage with peers and patients to investigate how their routine processes fit within the overall care system, and to explore potential improvements.

To support the conduct of improvement initiatives, project teams received training from CLAHRC NWL on improvement skills. Teams had limited prior experience and required encouragement and support to use quality improvement methods. Skills in team working and project management were also provided by CLAHRC NWL through on-going coaching and expert input.

One of the major features of complex systems is that they are self-organising. Healthcare professionals and patients are a critical resource to understand and effect change within complex systems, but for them to meaningfully engage requires training, support, resources and headroom in skills they can transfer to other implementation and improvement work [76, 77]. This learning is summarised in our substantive theory presented in Table 3.

## Discussion

SHIFT-Evidence provides a comprehensive overview of the challenges and corresponding actions required for successful implementation and improvement. These are summarised as three strategic principles and 12 ‘simple rules’. Exploration of the practical reality of making changes in frontline care settings reveals the need to reconceptualise the challenge of evidence translation to take account of system complexity.

### Systems evolve over time and have historical path dependencies

Our findings demonstrate that intervening in complex systems requires an understanding of the unique initial conditions (problems, opportunities, people, practices and patterns) in each local setting that are influenced by historical path dependencies. Scientific evidence about which interventions to use needs to be balanced with local system requirements, rather than assuming the starting point will be the same in each setting, and a commitment to continual improvement is needed to allow for the fact that systems evolve and adapt over time. This temporal dimension of systems thinking is reflected in the SHIFT-Evidence framework by the strategic principle ‘act scientifically and pragmatically’.

These findings challenge current conventions of seeing implementation as a one-off or time-limited activity, and build on Hawe et al.’s [61] proposal that interventions are ‘events in systems’. Further, a single pre-planned intervention, or set of interventions, is unlikely to be sufficient to achieve evidence implementation and improvements. Instead, multiple interventions are likely to be required; with the need emerging only as changes are implemented and system understanding grows. This builds on quality improvement approaches that promote iterative development over time [59, 78, 79], and organisational learning perspectives that value generative learning (e.g. double (and triple) loop learning) [73, 80].

We propose, in light of these findings, that terminology shifts from the use of the noun ‘intervention’ to the verb ‘intervening’. We believe that the concept of ‘intervening to achieve an improvement’ better reflects the iterative and negotiated process required to test multiple interventions whilst noticing and responding to local system requirements over an extended period of time (cf. Snowden’s probe-sense-response) [81].

### Systems are dynamic and interconnected

Interventions cannot be considered in isolation from the system they are implemented into. The uptake and effective utilisation of any specific intervention is dependent on established practices and processes of care. These practices and processes of care cannot be assumed to be working well, and often additional interventions will be necessary to address related and systemic problems. Intervening in complex systems requires an understanding of these dynamic and fluctuating processes. Understanding of system dynamics and interconnectivity is represented by the strategic principle ‘embrace complexity’.

This challenges current conventions of seeing interventions as bounded and discrete, and anticipating that such interventions will be used by people working in a rational linear manner. Interventions are inherently dependent on the context that they are used in, and it cannot be assumed that dependent processes and practices are working well. This builds on literature from operations management and patient safety in valuing an understanding of ‘work as is’ as opposed to ‘work as imagined’ [82, 83]; people in complex systems are challenged with making decisions in real-world conditions, under high pressure, with constrained time and resources, whilst balancing multiple priorities [84].

### Systems are made up of individual agents capable of self-organisation

The implications of systems evolving over time and their dynamic, interconnected nature are that capacity and capability needs to be built into the system to reflect, experiment and learn about intervening within the system over time. The strategic principle ‘engage and empower’ emphasises the critical role local system members play in identifying and solving local problems (although each person individually can only partially know or see the whole system), and the necessity for their willingness and motivation to adopt new ways of working.

This challenges current conventions of implementation activities being designed and conducted by people outside of the system, and draws attention to the unique insights provided by people within the local system (healthcare professionals, patients, managers) about how they self-organise and how they experience attempts to intervene. This builds on literature on co-production [65, 66, 85] and co-design [86] that emphasises the importance of engaging local stakeholders to solve problems that matter to them in their local setting and acknowledges the extensive work on understanding individual psychology of behaviour change and group dynamics [87, 88].

### Value of SHIFT-Evidence to practitioners and academics

SHIFT-Evidence is the first empirically grounded framework for evidence translation in complex systems that can help make predictions and provide explanations about challenges and influences on success.

This research adds to the complexity science literature, initially proposed by Plsek and Greenhalgh [28], describing healthcare as a complex system. Building on this perspective, it makes a unique contribution in considering the implications of complexity for deliberate attempts to intervene and introduce evidence-based practices [36, 37]. Our study focused on micro-level initiatives, but findings resonate with existing literature on complexity in relation to macro-level initiatives (e.g. policy, systems design) [29, 34]. By providing insights into the ‘sharp end’ of practice, SHIFT-Evidence can provide insights to policymakers and system leaders as to how ‘top down’ initiatives might be received in complex systems.

This study also contributes to literature on evidence translation and implementation. It advances research by Craig et al. [89] on complex interventions, and by McCormack et al. [90], amongst others, which recognised the importance of context in the uptake of evidence-based practices, and May et al. [38], which expanded their theory of implementation to consider context as a complex adaptive system. SHIFT-Evidence builds on these views to consider the interaction between interventions, implementation strategies and context as inseparable and interacting components of a complex system. This view is reinforced by complexity thinking by resisting the temptation to isolate or reduce a system to its component parts, and instead to take interest in the interactions and patterns that emerge across the whole system.

For academics, SHIFT-Evidence provides an explanatory and predictive framework. The substantive theory explains the challenges encountered during evidence translation in complex systems and provides a rationale for strategies and actions to overcome them. The ‘simple rules’ provide testable hypotheses about the actions conducive to success that can be tested through future research. In demonstrating the magnitude of the challenge faced, SHIFT-Evidence makes clear the need for interdisciplinary enquiries to advance understanding and practice.

For patients, practitioners, managers, policymakers and academics involved with designing, conducting or evaluating healthcare improvement initiatives, SHIFT-Evidence provides a common framework to guide their work and ensure they are considering the breadth of the practical realities of evidence translation and improvement. The strategic principles (‘act scientifically and pragmatically’, ‘embrace complexity’ and ‘engage and empower’) were designed to be intuitive, accessible and memorable. A common framework that represents the complex and dynamic nature of improvement should help practitioners, academics and patients collaborate more effectively to increase the likelihood of success. If practitioners and patients can easily access practical knowledge, they may be more willing to contribute to the creation of new knowledge and to participate in the design, conduct and evaluation of future change experiments. If researchers understand how their work directly helps practitioners achieve improvements, and influence the lives of patients, they may be more likely to produce outputs which in turn increase practitioner receptivity and access to research settings.

For policymakers, funders and senior managers, SHIFT-Evidence emphasises the significant investment required at all stages of improvement efforts, including providing frontline practitioners with the time to step back from their day-to-day activities and the support needed to overcome barriers and obstacles to improvement. Such resource commitment is often seen as a luxury rather than essential. By using this structured approach to support funding and prioritisation, this may allow optimal investment of available resources and disinvestment in initiatives that add little value.

### Limitations and future research

The quality of theory should be assessed by how useful it is in solving societal problems recognising that *“the published word is not the final one, but only a pause in the never-ending process of generating theory”* [91]. Therefore, rather than be seen as a finalised theory, or a perfect set of ‘simple rules’, the value of SHIFT-Evidence needs to be assessed through its usefulness in practice (and research), and should act as a catalyst for further improvement and refinement of the theory as predictions are tested.

The first limitation of this work is the transferability of the substantive (context-specific) theory to other settings beyond NWL and a UK cultural context. While research drew on a range of real-world improvement studies from different settings and on diverse clinical topics, all cases were from a single region (London, UK). Our wider author and team experiences suggest the cases represent wider national and global challenges (e.g. [92]). However, there is a need to explore the transferability of SHIFT-Evidence into other global settings and to continue to assess the comparative importance of the individual principles in different contexts.

The second limitation of this work is the transferability of findings to different intervention types and implementation and improvement approaches. All of the projects included in the empirical study were led by clinical leaders who voluntarily took on the role and defined the improvement area and evidence-based solutions, and in many instances this was done in collaboration with their teams and wider stakeholders. In addition, the use of a specific, quality improvement approach was promoted and supported in all project teams, although actual use of the approach was variable [93]. Further work is required to explore the transferability of SHIFT-Evidence to a greater diversity of intervention types (including organisational, system or policy level change) and implementation and improvement approaches.

The third limitation is methodological. The auto-ethnographic role of the researchers provided benefits including proximity to the subject matter, extensive contact with project teams and long-term relationships to explore how issues evolved over time. As all authors were senior members of the programme, there is a risk that their access to conversations and their perceptions and interpretations of the findings would be affected by status. The collaborative approach adopted between authors and other members of the CLAHRC NWL team (including more junior staff) allowed access to feedback from other programme participants and different types of conversations and ‘behind the scenes’ encounters. In addition, regular engagement with CLAHRC NWL project team members helped to triangulate findings and gain different perspectives. As such, the findings represent a culmination of discussion and sense making between the researchers and participants over an extended period of time. Evidence of these shared reflections exists in the publications co-authored with project teams that demonstrate insights into the challenges and complexity experienced (e.g. [94–96]). Interpretation of the results was further triangulated with other experts in the field and in analysis of extensive literature to support reflectivity and to increase the reliability and validity of the findings. However, further research is required to explore how different methodological or theoretical perspectives produce convergent or divergent findings.

Further research is required to examine how to effectively operationalise the SHIFT-Evidence ‘simple rules’ in practice [97]. For example, knowing that ‘understanding problems and opportunities’ is important does not provide detailed guidance on how to engage relevant stakeholders to access local knowledge nor how to make sense of complex system interactions. Many approaches, tools and methods for operations research [98, 99], network analysis [36], implementation and quality improvement have already been developed and studied [100–104], and this knowledge should inform the generation of structured and practical approaches that enable the SHIFT-Evidence ‘simple rules’ to be enacted in practice. It will also be necessary to work with advances in complexity sciences to develop new approaches to the practice and research of intervening in complex systems.

## Conclusion

SHIFT-Evidence is a unique framework with explanatory and predictive power grounded in the practical reality of evidence translation and improvement in healthcare. It advances thinking about how to intervene in complex systems, namely that, to achieve successful improvements from evidence translation in healthcare, it is necessary to ‘act scientifically and pragmatically’ whilst ‘embracing the complexity’ of the setting in which change takes place and ‘engaging and empowering’ those responsible for and affected by the change.

A series of 12 action-orientated ‘simple rules’ are proposed to guide patients, practitioners, managers, policymakers and academics to intervene in complex systems. We propose that efforts to translate evidence into practice should be reconceptualised from focusing on simple relationships between interventions and outcomes to understanding the complex and nuanced work required when ‘intervening to achieve an improvement’. This better reflects the iterative and negotiated process required to test multiple interventions whilst noticing and responding to learning that emerges from the system over an extended period of time.

## Box 1: A project narrative of evidence translation for community-acquired pneumonia (CAP)

This 18-month Collaboration for Leadership in Applied Health Research and Care (CLAHRC) North West London (NWL) project aimed to improve the timeliness and effectiveness of initial treatment of CAP during emergency hospital admission in order to improve patient outcomes and experience.

**Outline of problem (primarily explored during months 0–6)**

- Evidence-based treatment for CAP was identified by the project team through a review of the 137 national guideline recommendations [105]. Core recommendations requiring completion within 4 h of a patient arriving at hospital included oxygen assessment and treatment, measuring pneumonia severity and providing appropriate antibiotics.
- The project leads believed all clinicians were aware of the treatment guidelines. Doctors agreed they knew the evidence and were confident that they and their clinical teams were delivering high-quality evidence-based care; thus, the project was considered unnecessary by many senior clinicians.
- A baseline audit of local practice showed 0% of patients received all evidence-based care elements, with compliance ranging from 13% to 90% for the individual elements. Further investigation revealed that junior doctors’ awareness of evidence was lower than expected, and that doctors, pharmacists and nurses needed to coordinate their work within the few first hours of hospital admission.

**Initial solutions (tested and implemented during months 7–18)**

- An intervention was developed grouping the evidence-based care elements onto a single page paper-based ‘care bundle’ [106–108], designed to prompt action for all staff, including junior doctors, and coordinate care between professionals.
- The team collected weekly data on the extent to which each care element was delivered within 4 h. Following the initial implementation, low compliance persisted with < 5% of patients receiving all elements.
- To address poor uptake, the bundle was iterated 15 times over 12 months until the design and content was accepted by different clinical groups, the wording was clarified and the bundle deemed compatible with other usual care and documentation practices.
- During this initial phase it emerged that improvements needed to be addressed in the wider system, including updating oxygen and antibiotic prescribing policies and the lack of a process for ordering appropriate microbiological tests. Review of patient data also raised concerns about the accuracy of the initial diagnosis of CAP at first assessment.
- Four other sites across NWL engaged with the programme to adopt the CAP care bundle (cross site engagement started at month 12 of the original timeline, and continued for a further 18 months). New sites were motivated by data showing the care bundle improved delivery of evidence-based care but all spent several months appraising the evidence and intervention against their local experiences, knowledge, system and context before implementation commenced.

**Key learning about complexity**

- Coordinated solutions were required that involved different professions working with senior executives to change policies and overcome barriers. These actions, combined with better staff education and awareness of CAP and the care bundle, improved delivery of timely care.
- Despite initial success, many factors continued to threaten sustained success on the original site. Regular review of compliance data enabled factors causing variation to be identified and addressed. For example, when measures showed a sudden drop in compliance, the original team investigated and identified junior doctors’ rotation as a contributing factor. They devised ways to improve junior doctor training and awareness at induction.
- Staff from different sites met together and learned that their challenges were common. Much of the knowledge shared was tacit, and passed on through discussions rather than written or formalised knowledge exchange.
- Other sites experienced similar factors to the original site that influenced their sustained success, including staff turnover and the emergence of conflicting organisational improvement priorities.

**Outcomes**

- Although initially reluctant, rigorous weekly measurement allowed the team in the first site to track progress, identify potential improvements and, ultimately, to demonstrate success. Over 12 months, variation in delivery of the individual care elements reduced from 13–90% before the bundle to 74–92% afterwards, and overall compliance increased from 0% to 49% [109].
- Two sites achieved sustainable use of the bundle 1 year after the formal end of the project by integrating the bundle into the routine admission process. One site maintained measurement of CAP bundle compliance to continue monitoring and responding to variation in use and maintained high levels of compliance.

## Box 2: A project narrative of evidence translation in medicines management (MM)

The 18-monthCollaboration for Leadership in Applied Health Research and Care (CLAHRC) North West London (NWL) MM project aimed to implement an evidence-based post-discharge follow-up phone call [110, 111] to support patients whose medications had been changed during an emergency admission.

**Outline of problem (primarily explored during months 0–6)**

- The follow-up phone call intervention was intended to ensure patients understood their new medicines regimen. The project team expected the introduction of phone calls to be straightforward, but quickly discovered that they needed to address many related issues.
- Obtaining information about patients’ medication history to inform the follow-up phone call was a major problem, requiring triangulation of information from several sources after hospital admission. The availability of this information was recognised as a systemic problem which directly affected the ability to complete medicines reconciliation at discharge.
- Separate medication lists were maintained by up to four different professional groups for their own purpose (doctors, pharmacists, nurses and physiotherapists) with little awareness of each other’s documentation practices. This silo-working increased the risk of medication errors. For example, a patient with arthritis was unable to open bottles with a child-proof top. The physiotherapist was aware of this, but the pharmacy was not and continued to dispense medications in inaccessible containers.

**Initial solutions (tested and implemented during months 7–12)**

- Staff recognised that they would need to redesign the process, and renegotiate their roles to coordinate their work more effectively. A single agreed medicines reconciliation form was introduced, which allowed them to assess the quality of medicines reconciliation.
- Patients involved with the project challenged assumptions about relying on clinicians and organisations for this information. In a spin off project, clinical teams worked with patients to develop a patient-held ‘My Medications Passport’, which could act as an information source to support medicines reconciliation and help patients take greater ownership of their medication histories [112, 113].

**Key learning about complexity**

Investigation into the causes of medication errors revealed several variables affecting the process, including the number of patients being admitted, the complexity of each patient’s condition, and the number and type of medications per patient. These variables were further influenced by staff working practices, including the time available to reconcile an individual patient’s medications. Variation in doctors’ performance prompted the team to improve teaching for junior doctors emphasising the importance of documenting medication changes using standardised procedures for recording and reconciling medicines. Junior doctors assumed someone else completed the medication documentation, so the team worked with them until it was accepted as a routine responsibility.

- The team had to negotiate with the executive team to secure the appropriate budget and permission for the changes in medicine reconciliation to take place. Delivering longer term changes required permission or support from people outside the team, including the education leads responsible for doctors’ induction.
- Aligning the project to organisational priorities took time and effort, and helped secure vital resources, including executive support, to further champion the work and permission for team members to be released to support the project.
- At the start of the project, medicines reconciliation had poor visibility within the hospital and was not an organisational priority. The team worked to increase its profile, identifying how the work related to key hospital concerns, including the importance of medicines reconciliation to admissions avoidance, how it linked to the safe and effective flow of patients through emergency care, and how it contributed cost-savings by avoiding inappropriate prescribing.

**Outcomes**

During the project, the error rate in medicines reconciliation reduced from 24% to an average of 11%. Week-to-week variation reduced from 0%–74% to 0%–32% [94]; at this point the follow-up phone calls were re-instigated [114].

## Additional files

Additional file 1: Project and programme publications: a list of CLAHRC NWL projects conducted between 2008 and 2013, and supporting publications from the CLAHRC NWL programme (DOCX 32 kb)

Additional file 2: Research methods (DOCX 90 kb)

Additional file 3: Example of coding and theory development (DOCX 14 kb)

## Abbreviations

CAP: community-acquired pneumonia
CLAHRC: Collaboration for Leadership in Applied Health Research and Care
MM: medicines management
NIHR: National Institute of Health Research
NWL: Northwest London
SHIFT-Evidence: Successful Healthcare Improvement From Translating Evidence into complex systems
