# Supplementary material for: Simple rules for evidence translation in complex systems: A qualitative study
Source: BMC Med. 2018 Jun 20;16:92. doi: 10.1186/s12916-018-1076-9 (PMC6009041; doi:10.1186/s12916-018-1076-9)
Supplement: Supplementary file 1 — Project and programme publications: a list of CLAHRC NWL projects conducted between 2008 and 2013, and supporting publications from the CLAHRC NWL programme (DOCX 32 kb) [file 12916_2018_1076_MOESM1_ESM.docx]

S1 File.  Projects and Programme Publications

List of CLAHRC NWL projects conducted between 2008 -2013 and associated publications

During the first five years of CLAHRC NWL (2008-2013), 22 projects were conducted with 55 clinical teams over four annual rounds of projects (5, 7, 7 and 3, new projects commencing in Round 1-4 respectively (2009, 10, 11 and 12). Each new project lasted 18 months in duration and in many cases successful projects rolled out to new clinical teams after 12 months, providing a 6 month overlap between project rounds to support shared learning and peer-support between teams

| Project topic | Round | Description | Setting Type(s) | Number of sites | Publications |
| --- | --- | --- | --- | --- | --- |
| Adult Congenital Heart Disease | 4 | A patient-centred approach to improving advanced care planning for adults with congenital heart disease | Hospital | 1 | Swan, L. 2013. A cardiologist’s view on the palliation of congenital heart patients. European Journal of Palliative Care |
| Alcohol | 2, 3 | A screening-based approach to improving the early identification of harmful and hazardous alcohol use in primary and secondary care services (linked to financial incentives in primary care) | Hospital and Primary Care | 3 | Hamilton, F. 2013. Effect of financial incentives on delivery of alcohol screening and brief intervention (ASBI) in primary care: longitudinal study. *Journal of Public Health*;  Woodrow, SR. 2013.An identification and brief advice programme for low-risk alcohol consumption in an acute medical setting: an implementation study. *JRSM Short reports* |
| Ambulatory Care | 4 | A virtual ward based approach to improving patient access to effective ambulatory care services | Hospital | 2 |  |
| Anaesthetics |  | The use of real-time individualised feedback for anaesthetists to improve patient recovery following surgery | Hospital | 1 | Benn, J. 2012 Using quality indicators in anaesthesia: Feeding back data to improve care *British Journal of Anaesthesia*  Benn, J. 2013 What is normal or not? *British Journal of Anaesthesia*  D’Lima, D. 2017. Continuous monitoring and feedback of quality of recovery indicators for anaesthetists: a qualitative investigation of reported effects on professional behaviour. *British Journal of Anaesthesia* |
| Antibiotic Prescribing | 2 | The collection and sharing of routine prescribing data with staff to improve the quality and safety of antibiotic prescribing on acute wards | Hospital | 3 |  |
| Case Management | 1 | A protocol based approach to case management of patients with complex needs in primary care to improve integration and continuity of care | Primary Care | 2 | Thomas, P. 2010. Inter-organisational communication for patients being case managed by Community Matrons. *London Journal of Primary Care*;  Iliffe, S. 2011. Making a jigsaw puzzle in ten minutes: Is case management feasible in General Practice? *London Journal of Primary Care* |
| Children’s Allergies | 3 | A competency based approach to training to improve the care of children with allergic conditions | Hospital and Primary Care | 2 | Gore, C. 2016 New Patient Reported Experience Measure (PREM) for children with allergic disease. Development, validation and results from integrated care. *Archives of Disease in Childhood*  Warner, J. 2017 Integrating Care for Children with Allergic Diseases: UK experience *Current Allergy and Clinical Immunology* |
| Chronic Heart Failure | 2 | A clinical pathway approach to improving he diagnosis and management of heart failure and access to outpatient rehabilitation | Hospital | 2 |  |
| COPD Chronic Disease Management System | 3 | An electronic health record based approach to sharing the treatment plans for patients with COPD between primary and secondary care services | Hospital and Primary Care | 4 |  |
| COPD Discharge Care Bundle | 1,2,3,4 | A care bundle approach improving the delivery of evidence based care at discharge from hospital following an acute exacerbation of COPD | Hospital | 8 | Hopkinson, N. 2011. Designing and implementing a COPD discharge care bundle. *Thorax*;  Laverty, A. A. 2015. Impact of a COPD Discharge Care Bundle on Readmissions following Admission with Acute Exacerbation: Interrupted Time Series Analysis. *PLoS One*;  Lennox, L. 2014. Identifying the challenges and facilitators of implementing a COPD care bundle. *BMJ Open Respiratory Research*. |
| COPD Disease Telemonitoring | 2,3 | A technology based approach to delivering telemonitoring interventions to improve the care for patients with chronic respiratory disease and heart failure | Hospital and Community | 2 | Chatwin, M. 2016. Randomised crossover trial of telemonitoring in chronic respiratory patients (TeleCRAFT trial). *Thorax* |
| Community Acquired Pneumonia | 1,2,3 | A care bundle approach improving the delivery of evidence based care during emergency department attendance and admission to hospital for community acquired pneumonia | Hospital | 6 |  |
| Diabetes Prevention and Self-Management | 3 | A community based approach to developing local peer champions improving awareness of risk-factors for diabetes and promoting self-care | Public Health/Community | 1 |  |
| Diabetic Foot | 3 | A care bundle approach to improving the diagnosis and management of acute diabetic foot complications following admission to hospital | Hospital | 1 |  |
| HIV Testing | 1,2 | Use of point of care testing to improve the routine testing of HIV and serial CD4 counts of people in non-traditional settings such as the emergency department and mental health unit | Hospital and Mental Health Unit | 4 | CB Jones. 2012. Clinical Evaluation of the Determine HIV-1/2 Ag/Ab Combo test. *Journal of infectious diseases*;  Rayment, M. 2012. HIV testing in Non-Traditional Settings in the UK – the HINTS study: A multi-centre, prospective observational trial. *PLoS One*;  Thornton, AC. 2012 Exploring staff attitudes to routine HIV testing in non-traditional settings: a qualitative study in four healthcare facilities. *Sex Transm Infect* Sanger, C. 2013 Acceptability and necessity of HIV and other blood borne virus testing in psychiatric settings *British Journal of Psychology*" |
| Jaundiced Babies | 3 | A technology based approach to developing a new pathway to improve the diagnosis and management of jaundice in the maternity unit | Hospital and Community care | 2 |  |
| Medicines Management | 1,3 | Use of evidence-based medicines reconciliation/review form to improve medication management within the acute medical setting and during transfers of care | Hospital | 9 | Marvin, V. 2011. Phone-calls to a Hospital Medicines Information Helpline. Analysis of Queries from Members of the Public and Assessment for Harm from their Medicines. *International Journal of Pharmacy Practice*; Marvin, V. 2012. Improving Medicines Reconciliation and Communication at Transition of Care to and from an Acute Medical Unit. *Prescribing and Research in Medicines Management*;  Marvin, V. 2013. Pilot Study of the Use of a Medication review tool as an aid to stopping unnecessary medicines in older hospital patients. *Prescribing and Research in Medicines Management*  Abdul-Saheb, M. 2014 Intermediate Care for the Elderly *Geriatric Medicine*  Marvin, V. 2012 improving medicines reconciliation and communication at transition of care to and from an acute medical unit. *Pharmaco-epidemiology and Drug Safety*;  Barber, S. 2014. Evaluation of My Medication Passport: a patient-completed aide-memoire designed by patients, for patients, to help towards medicines optimisation. *BMJ Open*;  Duraisngham, S 2015 Stopping inappropriate medicines in the outpatient setting. *Geriatric Medicine*  Marvin , V. 2016 Applying quality improvement methods to address gaps in medicines reconciliation at transfers of care from an acute UK hospital *BMJ Open* |
| Pulmonary Rehabilitation | 4 | A patient-centred approach to improve information about pulmonary rehabilitation with the aim of increasing access, attendance and completion of PR services | Community Care |  | Jones, S. E. 2014. Pulmonary rehabilitation following hospitalisation for acute exacerbation of COPD: referrals, uptake and adherence. *Thorax* |
| Sickle Cell Anaemia | 2 | An education and training approach to improve the experience of people with sickle cell using primary care services | Primary Care | 1 | AlJuburi, G. 2012. Patients’ views on improving sickle cell disease management in primary care: focus group discussion. *JRSM Short reports*;  AlJuburi, G. 2012 Trends in hospital admissions for sickle cell disease in England, 2001/02-2009/10. *Journal of Public Health*;  AlJuburi, G. 2012. Views of patients about Sickle Cell Disease management in primary care: a questionnaire-based pilot study. *JRSM Short reports*;  AlJuburi, G. 2013. Socio-economic deprivation and risk of emergency readmission and inpatient mortality in people with sickle cell disease in England: observational study. *Journal of Public Health*;  Green, S. 2012. Characterising Emergency Admissions of Patients with Sickle Cell Crisis in NHS Brent: Observational study. *JRSM Short reports* |
| Stroke | 3 | Use of patient-centred information to improve patient knowledge and awareness of risk factors for stroke following admission due to stroke | Hospital | 1 |  |
| Vascular Risk Assessment | 2 | A screening-based approach to improving the early identification of cardiovascular disease risk in primary linked to financial incentives | Primary Care | 1 | Dalton, A 2010 Implementation of the NHS Health Checks programme: baseline assessment of risk factor recording in an urban culturally diverse setting. *Family Practice*  Artac, M. 2012 Assessment of cardiovascular risk factors prior to NHS Health Checks in an urban setting: cross-sectional study *JRSM Short Reports* |
| Wellbeing | 2,3,4 | Use of social marketing interventions and primary care link workers to improve access to psychological therapies for people from 'hard to reach' communities | Mental Health Trusts and Primary care | 2 | Evans, L. 2014 Improving patient and project outcomes using inter-organisational innovation, collaboration and co-design. *London Journal of Primary Care*;  Green, S. 2012. Mapping mental health service access: Achieving equity through quality improvement. *Journal of Public Health*;  Evans, L. 2014. Improving access to primary mental health services: Are Link Workers the answer? *London Journal of Primary Care*;  Poots, A. J. 2014 Improving mental health outcomes: achieving equity through quality improvement. *International* *Journal for Quality in Health Care* Green, S. 2015. A retrospective observational analysis to identify patient and treatment-related predictors of outcomes in a community mental health programme. *BMJ Open*; |

Other supporting publications produced from the CLAHRC NWL programme

| QI method development and use | |
| --- | --- |
| Plan-Do-Study-Act Cycles | - Taylor MJ. 2014.Systematic review of the application of the plan–do–study–act method to improve quality in healthcare. *BMJ Quality and Safety* - Reed JE. 2015, The problem with Plan-Do-Study-Act cycles. *BMJ Quality and Safety* - Reed, J 2016 The foundations of quality improvement science. *Future Healthcare Journal* |
| Measurement for Improvement | - Curcin, V. 2010 Towards a scientific workflow methodology for primary care database studies. *Stat Methods Med Res* - Doyle, C. 2010 Understanding what matters to patients – identifying key patients' perceptions of quality. *Journal of the Royal Society of Medicine Short Reports* - Curcin, V. 2014 Model-driven approach to data collection and reporting for quality improvement *Journal of Biomedical Informatics* - Lovett, D.2014 Using geographical information systems and cartograms as a health service quality improvement tool *Spatial and spatio-temporal epidemiology* - Poots, A. 2012 Statistical process control for data without inherent order *BMC Medical Informatics and Decision Making* - Portela MC,. 2015. How to study improvement interventions: a brief overview of possible study types. *BMJ Quality and Safety* - Etchells E, 2017. Value of small sample sizes in rapid-cycle quality improvement projects 2: assessing fidelity of implementation for improvement interventions. *BMJ Quality and Safety* - Poots, A. 2017 How to attribute causality in quality improvement: lessons from epidemiology. *BMJ Quality & Safety* |
| Process Mapping | - Antonacci G. 2018.The use of process mapping in healthcare quality improvement projects. *Health Services Management Research* |
| Patient and Public Involvement & Engagement | - Matthews, R 2010 Involving patients in service improvement. *Nursing Management* - Matthews, R. 2012. Patient experience as a dimension of quality and nursing practice. *British Journal of Cardiac Nursing* - Taylor, M 2013 Using Virtual Worlds for Patient and Public Engagement *International Journal of Technology, Knowledge and Society* - Pizzo, E. 2014 Patient and Public Involvement: How much do we spend and what are the benefits? *Health Expectations* - Ocloo J. 2016. From tokenism to empowerment: progressing patient and public involvement in healthcare improvement. *BMJ Quality and Safety* |
| Stakeholder Engagement | - Reed JE. Eliminate slogans and remove barriers to pride in work. 2016. *Journal of Health Service Research and Policy* |
| Sustaining Improvement | - Doyle C, Howe C, Woodcock T et al. 2013. Making change last: applying the NHS institute for innovation and improvement sustainability model to healthcare improvement. *Implementation Science* - Lennox L.. 2017. What makes a sustainability tool valuable, practical and useful in real-world healthcare practice? A mixed-methods study on the development of the Long Term Success Tool in Northwest London. *BMJ Open* - Lennox L, Maher L, Reed JE. 2018. Navigating the sustainability landscape: A systematic review of sustainability models. *Implementation Science* |
| Action Effect Diagrams | - Reed JE. 2015. Designing quality improvement initiatives: the action effect method, a structured approach to identifying and articulating programme theory. *BMJ Quality and Safety* - Issen, L. 2018. Criteria for evaluating programme theory diagrams in quality improvement initiatives: a structured method for appraisal. *International Journal for Quality in Health Care* |
| QI Education | - Myron R. 2017. Professionals learning together with patients: An exploratory study of a collaborative learning Fellowship programme for healthcare improvement. *Journal of Interprofessional Care* |
| Literature reviews and view points | - Reed, J 2011 Centres for Healthcare Improvement: solution to the quality problem *Journal of the Royal Society Medicine* - Doyle, C. 2012 A review of evidence on the links between patient experience and clinical safety and effectiveness *BMJ Open* - Reed, J. 2013 Making an impact? The emergence of Improvement Science in healthcare. *International Journal of Science in Society* |

| Evaluations | |
| --- | --- |
| Cross project evaluations | - Howe, C. 2013 Supporting improvement in a quality collaborative *British Journal of Healthcare Management* - Howe, C. *2014.*  Improving engagement in a quality collaborative. *British Journal of Healthcare Management* - Renedo, A. 2015. Spaces for citizen involvement in healthcare: an ethnographic study. *Sociology*Green, S et al. 2017 Identification of factors that support successful implementation of care bundles in the acute medical setting: a qualitative study. *BMC Health Services Research* - Green, S et al. 2018. Co-designing interventions in quality improvement initiatives: Notes from the field. *Journal of Health Design* |
| External evaluations of CLAHRC NWL | - Caldwell, S. 2012. Studying policy implementation using a macro, meso and micro frame analysis: the case of the Collaboration for Leadership in Applied Health Research & Care (CLAHRC) programme nationally and in North West London. *Health Research Policy and Systems* - Renedo, A. 2011. Healthcare professionals' representations of ‘patient and public involvement’ and creation of ‘public participant’ identities: Implications for the development of inclusive and bottom‐up community participation initiatives*. Journal of Community & Applied Social Psychology* - Pizzo, E. 2014. Patient and public involvement: how much do we spend and what are the benefits? *Health Expectations* - Spyridonidis, D. 2014. Understanding hybrid roles: the role of identity processes amongst physicians. *Public Administration* - Renedo A. 2015. Patient and Public Involvement in Healthcare Quality Improvement: How organizations can help patients and professionals to collaborate. *Public Management Review* - Renedo, A*.* 2015. Developing patient-centred care: an ethnographic study of patient perceptions and influence on quality improvement. *BMC Health Services Research* - Spyridonidis, D. 2015. Leadership for Knowledge Translation: The Case of CLAHRCs. *Qualitative Health Research* - Filipe A. 2017 The co-production of what? Knowledge, values, and social relations in health care *PLOS Biology* |
| Evaluations for national CLAHRC Programme (including NWL) | - Martin, G. 2011 The challenges of evaluating large-scale, multi-partner programmes: the case of NIHR CLAHRCs *Evidence and Policy* - Currie, G 2013 From what we know to what we do: lessons learned from the translational CLAHRC initiative in England *Journal of Health Services Research & Policy* - Oborn E. 2013 Balancing exploration and exploitation in transferring research into practice: a comparison of five knowledge translation entity archetypes. *Implementation Science* - Evans, S. 2014. Supporting knowledge translation through collaborative translational research initiatives: ‘Bridging’ versus ‘blurring’ boundary-spanning approaches in the UK CLAHRC initiative. *Social Science and Medicine* - Lockett A. 2014 A formative evaluation of Collaboration for Leadership in Applied Health Research and Care (CLAHRC): institutional entrepreneurship for service innovation. *Health Services and Delivery Research* |
