# Supplementary material for: Simple rules for evidence translation in complex systems: A qualitative study
Source: BMC Med. 2018 Jun 20;16:92. doi: 10.1186/s12916-018-1076-9 (PMC6009041; doi:10.1186/s12916-018-1076-9)
Supplement: Supplementary file 2 — Research methods (DOCX 90 kb) [file 12916_2018_1076_MOESM2_ESM.docx]

S2 File. Research Methods

This appendix provides additional details on the methods used including details of ethics, the researchers, data collection, coding and grounded theory development.

# Ethics

All CLAHRC NWL projects discussed in this manuscript independently applied for ethics e.g.[1] or obtained ethics waivers e.g. [2]

The independent evaluation of CLAHRC NWL by Imperial College Business School was approved by Central London Research Ethics Committee (REC approval number 09/H0718/35).[3]

Information from these studies were reviewed using secondary analysis to inform the research presented in this paper.

In line with precedent for auto-ethnographic observations in organisations further ethical approval was not obtained for the research presented in this paper (e.g. [4-6]). Field permissions were obtained from project teams and organisational leaders at CLAHRC NWL.

This decision was informed by careful consideration of the ethical dimensions of this work:[7]

i. Participants: Only NHS staff, project team members and CLAHRC NWL were involved in this research. No patients or patient indefinable information were included.

ii. Complete Member Researchers: The role of the authors as auto-ethnographers meant that no additional data was collected outside of normal working practices and the act of reflecting on and learning from experiences within the programme was a core component of the author’s roles in leading a learning organisation.

iii. Consent: All project teams had provided written consented to be part of the CLAHRC NWL programme and for their projects to be part of a cross-project analysis to advance understanding of how to translate evidence into practice. Organisational consent was implicit as the the Director of the CLAHRC NWL programme is an author. Project teams were reminded regularly of the research taking place, including at 6 monthly project reviews and at 3 monthly collaborative learning events where emergent findings were presented and discussed.

iv. Privacy: Data analysis was conducted so that coding was not identifiable to individuals or teams, and only abstract themes which emerged from cross project analysis are presented. Written consent has been provided for the two case studies by the two clinical leads of the associated project teams, who have approved the text included in the manuscript.

# Researchers

The CLAHRC NWL programme had a core team of researchers, managers, improvement advisors, data analysts and other roles who provided support and guidance to the conduct and evaluation of projects. The four authors of this paper were all members of this core team which increased in size from 14 members in 2009 to 24 members in 2013.

From 2008-2013 JR was Head of Research Strategy for CLAHRC NWL. She was involved in writing the original CLAHRC NWL funding application with DB and played a lead role in establishing the programme. Her role included oversight and strategic management of all research activities within the CLAHRC NWL programme. In addition she was regularly involved in acting in an advisory role to the CLAHRC NWL core team and project teams. She participated in daily and weekly group meetings with members of the CLAHRC NWL team and individual 1:1 meetings, and regularly met with project teams either informally, in formal meetings, or in education or advisory sessions. Her prior experience in the NHS was in research management, and before that JR had gained her PhD in an academic chemistry laboratory. JR brought a fresh perspective to make sense of the practical reality of evidence translation and improvement, whilst having a deep understanding of the scientific nature of empirical studies.

CH joined CLAHRC NWL in 2010 as programme lead for the acute care theme. CH had oversight of all projects working in the theme (approximately half of total projects) and worked directly with project teams on a daily basis. Out of all of the authors she had the most depth of interaction with the project teams. CH also participated in daily and weekly meetings with the CLAHRC NWL core team where progress, challenges and opportunities were discussed and reflected upon. CH had experience in using quality improvement approaches and had worked as a manager in the NHS for 10 years. She brought an in-depth understanding of quality improvement approaches and organisational perspectives, and insights and experience from previous improvement efforts to triangulate with our experience in CLAHRC NWL.

CD joined CLAHRC NWL in 2009 as the programme lead for evaluation. Of all the authors CD had the most formalised researcher role and was responsible for literature reviews, supporting project evaluations and cross project research. He worked closely with project teams, CLAHRC NWL core team and evaluation partners to support aspects of research and evaluation. His interactions with the project teams tended to be more formal than the other authors and would most commonly interact with teams in formal review meetings or educational settings, although he did build close working relationships with project teams where he was supporting evaluation activities. His previous experience had been working as a researcher in healthcare evaluations. Although CD was the author furthest removed from the daily activities of the project teams, he was well positioned to provide valuable insights on progress made over time, emerging trends and themes across project teams, and connections to the wider academic literature.

DB led the original funding application in collaboration with JR and was appointed director of the overall programme. DB had oversight of all aspects of the CLAHRC NWL programme and brought with him a wealth of experience from being a practicing clinician and medical director, and leadership of and involvement with national and international quality improvement initiatives. DB was closely involved with the progress of the CLAHRC NWL projects and played a dual role of both advising and building capability in the core CLAHRC NWL team and project teams, and where appropriate challenging and critiquing progress made in order to hold accountability for the research funding. Through drawing on extensive experience and knowledge of the literature and practice DB regularly provided insights to make sense of and interpret our experience and maintained a clear focus on the practical needs of those involved in conducting evidence translation and improvement as a focal point for our work.

# Data collection

Data was collated from written documentation collected for operational and research purposes including from the project teams, CLAHRC NWL core team and external evaluators. Collation was facilitated by shared core team file storage and an online tool (Web Support for Improvement in Healthcare) for capturing project-level data (Appendix 2).[8]

The authors acted as auto-ethnographers drawing on their lived experiences in running and researching the CLAHRC NWL programme. Analytical autoethnography refers to research in which the researcher is (1) a full member in the research group or setting, (2) visible as such a member in published texts, and (3) committed to developing theoretical understandings of broader social phenomena. The benefits of an ethnographic approach to gathering data included direct access to and observation of actions, events, scenes and people in real-time over a 5 year period, with opportunities to follow up on emergent patterns and problems.[9, 10]

## Documentation

Documentation from project teams included structured summary documents (original project proposals, 6 month progress reviews, and final project reports), project posters and power-point presentations given by the teams, and documentation resulting from the use of quality improvement methods including programme theory diagrams, process mapping, plan-do-study-act cycles, measurement for improvement, NHS Sustainability Model and stakeholder maps. In addition many of the teams published peer-reviewed or grey literature accounts of their projects ranging from narrative accounts to formal outcome evaluation.[11-19] Collation was facilitated by shared core team file storage and an online tool (Web Support for Improvement in Healthcare) for capturing project-level data.[8]

Documentation from CLAHRC NWL core team included notes and minutes taken at meetings and meetings between the core-team and project teams, and risk and learning logs maintained through weekly progress review meetings. Peer-reviewed papers were published by the core team on use of quality improvement methods. [20-22] [8, 23-26] [27, 28]

Three external evaluations of the CLAHRC NWL programme (including the role of leadership, patient and public involvement, and development of CLAHRC NWL as an organisation) took place. Documentation from evaluations conducted by external partners included formative feedback reports that were provided to the core team on 6 monthly basis. Results from these evaluations have been published elsewhere including social science perspectives on patient engagement and context for collaborative working [29-32]; organisational science and policy perspectives on the overall CLAHRC NWL programme.[33-38]

## Autoethnography

Data collection, theoretical sampling and the constant comparative analysis of data was supported by the authors’ roles as programme participants.[9, 39] In line with Anderson’s definition we adopted an analytic autoethnographic approach in recognition that we were conducting ethnography of our “own people” rather than ethnography of self (emotive autoethnography).[10]

As described above, all authors had formal roles in CLAHRC NWL core team and therefore had complete member research status. As the programme progressed the need to build on informal cross-project learning about the practical reality of evidence translation and improvement became apparent. The authors recognised the unique vantage point their roles provided them, including the depth of insights gained through this lived experience and close working relationships with the project teams. This role as autoethnographers was in part opportunistic, responding to the emergent learning from the programme, and in part covert; the original programme aim had been to advance knowledge related to evidence translation, but the exact nature of the enquiry did not emerge until the programme was underway. The research approach increased in formality over time as the clear purpose of the research emerged and formal coding began.

The benefits of ethnographic approach to gathering data in grounded theory:[9]

- Direct access to events, scenes and people (both to project teams, those in CLAHRC NWL core team who played a support or advisory role to these teams, and to those conducting formal evaluations of the programme or aspects of it)
- Participation over time (over 5 years of original funding period)
- Observation of actions and events in real time (regular interactions with teams over the life of a project provided insight into how events unfolded over course of project and in particular how strengths or challenges identified early in project life time were acted upon and the influence this had on project progress)
- Opportunities to follow up on emergent patterns and problems (each new round of projects provided an iterative opportunity to test emergent codes and categories in increasingly diverse settings and situations. This acted as a constant ‘reality check’ to support the process of induction, deduction and verification during constant comparative analysis)

The authors collectively conducted thousands of hours of participant observation through daily interaction with project team and core team members, which included formal and informal meetings and site visits as well as involvement in day-to-day project activities. Throughout the programme weekly meetings were held between the authors and other core team members to reflect on the progress of project teams and barriers and facilitators they were encountering, how challenges could be or were being overcome, and what lessons had been learnt. In addition, regular project and programme reviews were held which acted as informal focus groups to explore emerging issues and understand different stakeholder perspectives. Quarterly collaborative learning events provided an opportunity for interaction and shared learning between project teams and with the CLAHRC core team. Observation and meeting notes were kept both by individual authors and in central resources available to all in the core team such as risk and learning logs.

# Literature review

Building on literature that influenced the initial design of the CLAHRC NWL programme[40-43], extensive literature review was conducted over the life of the programme as relevant literature was identified through snowball searches and expert academic advice. Topics covered in this search process include knowledge translation (e.g. [44-46]) , implementation (e.g. [47-50]) and quality improvement (e.g. [43, 51-61]) , as well as other areas including complexity science (e.g. [62-65]) organisational and management studies (e.g. [66-74], patient safety (e.g. [75-77]education (e.g. [78-81]) , behavioural sciences (e.g.[82-84] , statistics (e.g. [85, 86]) and philosophy of science (e.g. [87-89]).

Where possible existing systematic reviews and established frameworks or models were reviewed, and seminal papers or books were also referred to, with the aim of understanding the origins of work and world views. For example, review of the literature relating to Knowledge Translation identified recent reviews on the topic (e.g. [90, 91]), and from these sources we were led to review earlier literature and seminal papers in the field, some of which we were already familiar with (e.g organisational learning[66]). and others that were new to us, therefore opening door to learning from other fields (e.g. absorptive capacity[92]) which in turn led to identification of other key works that were explored and incorporated into our sense making process.

This process was conducted in parallel to the running of the programme and conduct of research and grounded theory analysis. New literature was explored to help explain the challenges we experienced in translating evidence into practice. Key concepts were embraced to help explain and provide insights into our experiences (e.g. communities of practice theory[72] was found to be of use to help make sense of the challenges experienced in bringing together professionals from different backgrounds to work collaboratively on a common project), and gaps in the existing literature were identified (e.g. whilst current literature is strong in describing the existence and characteristics of communities of practice, there is less knowledge available about how to “create” new, and in particular, multidisciplinary, communities of practice – insights that would have been helpful to inform the daily challenges experienced by the programme). Further details of how literature review informed theory development are provided in the “reconceptualisation” section below.

# Grounded Theory Approach

This study used grounded theory to generate a theoretical explanation of evidence translation and improvement in healthcare from empirical data.[39, 93] Our approach was informed by a pragmatist perspective which understands that reality is made by and experienced by human actions and interactions, and places an emphasis on temporality and the process by which events unfold.[94, 95] The quality of pragmatist theory should be assessed by how useful it is in solving societal problems recognising that *“the published word is not the final one, but only a pause in the never-ending process of generating theory”*.[93]

Referencing the description of the approach by Glaser and Strauss[93] we present our methods in line with its three key concepts: “constant comparison,” in which data are collected and analysed simultaneously; “theoretical sampling,” in which decisions about which data should be collected next are determined by the theory that is being constructed: and “theoretical sensitivity” reflecting the importance of interpretive insight leading to reconceptualization and abduction. [96]

## Coding and constant comparative analysis

Three coding stages were followed; open, axial and selective coding.[94]

Initial open coding built an empirical understanding of the practical reality of those conducting evidence translation and improvement, the barriers and challenges they encountered and how they were able to overcome them. Empirical generalisations were then derived and reviewed in constant comparative analysis with relevant constructs from established theories, frameworks and the wider literature (described above and in the “reconceptualisation” section below)

Axial coding identified relationships between the barriers, facilitators and challenges to evidence translation and improvement and how the strategies adopted and actions taken by project teams influenced project success.

Initial categories were organised to identify relationships (axial coding) between the following concepts:[39, 94, 97]

1. the phenomenon under study (the translation of evidence into practice to achieve improvements in care)
2. the conditions related to that phenomenon (understanding the context in which such changes took place and how this presented as barriers, challenges or facilitators to translation or improvement);
3. the actions and interactional strategies directed at managing or handling the phenomenon (the actions taken or strategies adopted by CLAHRC NWL project teams as they approached evidence translation and improvement) and;
4. the consequences of the actions/interactions related to the phenomenon (the degree of success or otherwise that teams achieved in the process and outcomes of translation and improvement attempts).

As our study was particularly focused on the need to provide guidance to practitioners, the potential practical utility of the emerging theory was a priority; it needed to be able to guide future attempts at solving similar problems, and to explain and hypothesise about the actions required to support successful translation and improvement. Therefore we paid close attention to relationships between actions and strategies that, if taken, had a positive effect on project progress and outcomes, or if they were absent or overlooked were observed to have a detrimental impact. This informed the development of “simple rules” that represented the findings of our substantive theory.

Selective coding was then used to identify a core ‘focal-point’ category for the development of a conceptual framework making explicit the relationship between the substantive theoretical categories.

## Theoretical sampling in response to emergent analysis

Decisions about which data should be collected were driven in part by the embedded nature of the researchers (as auto-ethnographers) and by emergent themes from constant comparative analysis. The authors had the rare luxury of being fully immersed in the daily running of the programme and data collection processes. This constant immersion in the data has been described as akin to “drowning”.[98] In the authors respect this immersion often felt like regularly being “hit by tidal waves” as new insights and revelations emerged rapidly and often unexpectedly, challenging our existing assumptions and expectations.

In this respect theoretical sampling was in part deliberate (driven by the authors curiosity to explore further categories and themes that emerged from analysis in more detail), and in part opportunistic (driven by the organic nature of the immersion experience, with new and unexpected themes presenting themselves and demanding to be further explored). An example of this opportunism and unexpected learning is when projects conducted in Round 1 were intended to be “rolled out” to other sites in Round 2. The experience of “roll out” raised many new an unexpected questions which became key themes to inform future analysis (e.g. the reluctance of sites to take up ideas from elsewhere, the need to repeat the same processes of evidence review and intervention review that took place in the initial site, the importance of working slowly through stages of understanding local problems and creating buy-in to a new change idea, and the diversity of problems with “usual care” that different sites had to deal with). Thus our theoretical sampling was driven by growing realisations that emerged from the data, and by unexpected programme events that forced realisation upon us. As such we were constantly alert to and surprised by unanticipated developments that we were able to incorporate into our sampling approach and data analysis strategy.

The iterative process of constant comparative analysis and theoretical sampling (deliberate and opportunistic) was conducted within each round (identifying common themes and categories across projects running in parallel), and between the four rounds (developing and testing themes and categories overtime). The diversity of projects sampled meant that examples of both positive instances, where teams were able to achieve successful implementation or improvement or overcome particular barriers or challenges, and negative instances, where teams had limited success.

Overtime our observations produced less surprises, and the experiences of the project teams started to become more predictable (in line with our emerging theory). We started testing strategies to anticipate and respond to the challenges of evidence translation and improvement. What at one point had been overwhelming and unexpected, became foreseeable and in some ways manageable.

## Reconceptualisation

Empirical generalisations were constantly compared with relevant constructs from established theories, frameworks and the wider literature to help inform or explain our experiences and to enrich our thinking as coding and categories developed. A two-way process ensued between reviewing and drawing learning from established theories, and critically analysing existing literature in light of our experiences and emerging conceptualisation. At the beginning literature was looked at to provide explanations and theories to help make sense of our experiences. Over time this changed, as our theory developed and conceptual categories emerged from the “daily realities” of our experience we started to revisit the literature with a more critical eye, attempting to “make sense” of our findings that did not “fit” with established theories and perspectives.

As category saturation emerged and our conceptualisation developed we embarked on a more formal comparative analysis of the literature, deliberating contrasting our emerging conceptual framework with established frameworks from fields of knowledge translation, implementation and quality improvement. This formal comparative analysis helped drive the sense making process by forcing an explicit consideration of differences and similarities with other frameworks, and helped improve our articulation of what our key learning was. The formal comparative analysis is in preparation for publication elsewhere.[99]

The process of emersion, deliberate and opportunistic theoretical sampling, and constant comparative analysis between the data and existing theories and frameworks led to many “light bulb moments” about the nuanced differences between our conceptualisations and established perspectives. These included, but are not limited to:

- Intervention focus versus problems with usual care: At the beginning we had assumed the basic practices and processes of care would be working well and wouldn’t require any additional work. Realising that this wasn’t the case (not just once, but over and over again) challenged our perceptions of the work that was required to embed evidence and improve care.
- Everything was much more complex than anticipated, and took a lot longer to resolve: The desire to move quickly and “make a difference” was countered by the careful patience and negotiation required to work slowly towards a shared understanding and perspective. Time for (and commitment to) two way learning was critical. Many concerns turned out to be valid and required exploration and resolution. Simply telling people what to do, or showing the evidence, was not enough to drive change.
- Implementation versus improvement: We had started with the assumption that we were implementing evidence based practices to improve care. Our learning demonstrated that one does not equal the other, and that often many other issues require addressing to improve quality of care beyond or as a prerequisite to the evidence based intervention of interest. Analysis of the literature led us to recognise a fundamental divide between implementation, where the intervention is of primary interest, and improvement, where achieving a goal is of primary interest. This subtle but significant difference helped us better make sense of our place within the literature.
- Pragmatism versus realism: Underlying the tension between implementation and improvement literature we identified a deeper and more profound disconnect in ontological and epistemological outlooks. Implementation science (and evidence based medicine more generally) tend to focus on generating generalizable knowledge about the success (or otherwise) of interventions to achieve predefined outcomes – aiming to generate positivist or realist statements about causality. Improvement science tends to focus more on processes for context driven problem solving, with the perception that local learning needs to take place, and local context navigated, in order to achieve meaningful change in a local setting, with less emphasis on production of generalizable or transferable knowledge – aligning to a more pragmatist view of science to solve local problems. This difference helped us to make sense of different literature, and different expectations and experiences of project team and programme members.
- Complexity: Our final “ah-ha” moment was the realising the essential implications of working in complex systems. We had been aware of complexity literature from an early stage of the programme, but had not fully appreciated how it helped explain our findings (why translating evidence into practice to achieve improvements is so nuanced, locally dependent, and hard work!). Applying complexity as a predominant lens to make sense of and interpret our data felt like our research had “come home”. Rather than trying to position ourselves in literature that had limited awareness of complexity or the implications of working in complex systems, we were finally empowered with a language and world view that helped make sense of our experiences, and allowed us to explain them to others in a more meaningful way.

After several rounds of theoretical sampling and constant comparative analysis - and many “ah-ha moments” - a substantive theory was proposed that we felt was “faithful to the everyday realities” of evidence translation and improvement.[94] This was shortly after the start of Round 4 (42 months since programme launch). The remainder of the programme provided an opportunity for further constant comparative analysis with new project teams to support verification of our theory through reflection on the accuracy, relevance and utility of the categories and to further test the interchangeability of indicators.[100] During this phase the initial substantive theory was presented and discussed on a number of occasions within the core team and with the wider CLAHRC NWL community of academics, practitioners and patients at events, meetings and workshops, and with external experts. As a result of further constant comparative analysis and conversations, modifications were made to the language, descriptions and presentation of the categories but no substantial changes to the underpinning concepts suggesting that saturation had been achieved, supporting the premise and validity of the substantive theory. For example, in several iterations the 12 ‘simple rules’ were felt to underpin four strategic principles. The fourth principle “invest in continuous improvement” was uncomfortable in that the 3 underpinning simple rules did not feel cohesive. Constant revisiting of this discomfort and re-analysis of the nature of the 3 underpinning rules convinced us that continuous improvement was in fact an integral part of the temporal process of *acting scientifically and pragmatically*, and the others were features of complexity and agency. So the final three strategic principles were determined.

An example of coding development is included in Appendix 3.

# References

1. Marvin V, Park C, Vaughan L, Valentine J: **Phone-calls to a Hospital Medicines Information Helpline. Analysis of Queries from Members of the Public and Assessment for Harm from their Medicines**. *International Journal of Pharmacy Practice* 2011, **19**(2):115-122.

2. Hopkinson NS, Englebretsen C, Cooley N, Kennie K, Lim M, Woodcock T, Laverty AA, Wilson S, Elkin SL, Caneja C *et al*: **Designing and implementing a COPD discharge care bundle**. *Thorax* 2012, **67**(1):90-92.

3. Spyridonidis D, Hendy J, Barlow J: **Leadership for Knowledge Translation The Case of CLAHRCs**. *Qualitative Health Research* 2015:1049732315583268.

4. Peck E: **The Performance of an NHS Trust Board: Actors' Accounts, Minutes and Observation**. *British Journal of Management* 1995, **6**(2):135-156.

5. Parker LD: **Boardroom Operational and Financial Control: an Insider View***. *British Journal of Management* 2008, **19**(1):65-88.

6. Pugliese A, Nicholson G, Bezemer P-J: **An Observational Analysis of the Impact of Board Dynamics and Directors' Participation on Perceived Board Effectiveness**. *British Journal of Management* 2015, **26**(1):1-25.

7. Tolich M: **A critique of current practice: Ten foundational guidelines for autoethnographers**. *Qualitative Health Research* 2010, **20**(12):1599-1610.

8. Curcin V, Woodcock T, Poots A, Majeed A, Bell D: **Model-driven approach to data collection and reporting for quality improvement**. *Journal of Biomedical Informatics* 2014, **52**:151-162.

9. Charmaz K: **Constructing grounded theory: A practical guide through qualitative analysis (Introducing Qualitative Methods Series)**. 2006.

10. Anderson L: **Analytic autoethnography**. *Journal of contemporary ethnography* 2006, **35**(4):373-395.

11. Hopkinson N, Englebretsen C, Cooley N, Kennie K, Lim M, Woodcock T, Laverty A, Wilson S, Elkin S, Caneja C *et al*: **Designing and implementing a COPD discharge care bundle**. *Thorax* 2011.

12. Laverty AA, Elkin SL, Watt HC, Millett C, Restrick LJ, Williams S, Bell D, Hopkinson NS: **Impact of a COPD Discharge Care Bundle on Readmissions following Admission with Acute Exacerbation: Interrupted Time Series Analysis**. *Plos One* 2015, **10**(2).

13. Green S, Poots A, Marcano-Belisario J, Samarasundera E, Green J, Honeybourne E, Barnes R: **Mapping mental health service access: Achieving equity through quality improvement**. *Journal of Public Health* 2012.

14. Benn J, Arnold G, Wei I, Riley C, Aleva F: **Using quality indicators in anaesthesia: Feeding back data to improve care**. *British Journal of Anaesthesia* 2012, **109**(1):80-91.

15. Green S, Phekoo K, Grover V, Lovenduski J, Anderson M, Bowden-Jones O, Foxton M: **Implementing an Identification and Brief Advice Programme for Hazardous Alcohol Consumption in an Acute Medical Setting**. *Alcohol and Alcoholism* 2012.

16. Hamilton F, Laverty A, Gluvajic D, Huckvale K, Car J, Majeed A, Millett C: **Effect of financial incentives on delivery of alcohol screening and brief intervention (ASBI) in primary care: longitudinal study**. *Journal of Public Health* 2013.

17. Hamilton F, Laverty A, Vamos E, Majeed A, Millett C: **Effect of financial incentives on ethnic disparities in smoking cessation interventions in primary care: cross sectional study**. *Journal of Public Health* 2012.

18. Doyle C, Lennox L, Bell D: **A review of evidence on the links between patient experience and clinical safety and effectiveness**. *BMJ Open* 2012.

19. Rayment M, Thornton A, Mandalia S, Elam G, Atkins M, Jones R, Nardone A, Roberts P, Tenant-Flowers M, Anderson J *et al*: **HIV testing in Non-Traditional Settings in the UK – the HINTS study: A multi-centre, prospective observational trial**. *PLoS One* 2012, **7**(6):e39530.

20. Reed J, McNicholas C, Woodcock T, Issen L, Bell D: **Designing quality improvement initiatives: the action effect method, a structured approach to identifying and articulating programme theory**. *BMJ Quality and Safety* 2014, **23**:1040-1048.

21. Taylor M, McNicholas C, Nicolay C, Darzi A, Bell D, Reed J: **Systematic Review of the Application of Plan-Do-Study-Act Method to Improve Quality in Healthcare**. 2013.

22. Doyle C, Howe C, Woodcock T, Myron R, Phekoo K, McNicholas C, Saffer J, Bell D: **Making change last: applying the NHS institute for innovation and improvement sustainability model to healthcare improvement**. *Implementation Science* 2013, **8**(1):127.

23. Doyle C, Reed J, Woodcock T, Bell D: **Understanding what matters to patients – identifying key patients' perceptions of quality.** *Journal of the Royal Society of Medicine Short Reports* 2010, **1**(13):1-6.

24. Poots A, Woodcock T: **Statistical process control for data without inherent order**. *BMC Medical Informatics and Decision Making* 2012, **12**(86).

25. Doyle C, Jones M, Bell D: **Monitoring patient flow in emergency departments; the use of scatterplots vs time based measures to assess patient flow in A&E**. *European Journal of Emergency Medicine* 2013.

26. Woodcock T, Poots A, Bell D: **The impact of changing the 4 h emergency access standard on patient waiting times in Emergency Departments in England**. *Emergency Medicine Journal* 2012.

27. Howe C, Bell D: **Improving Engagement in a Quality Collaborative**. *British Journal of Healthcare Management* 2014, **20**(11):528-535.

28. Howe C, Randall K, Chalkley S, Bell D: **Supporting improvement in a quality collaborative**. *British Journal of Healthcare Management* 2013, **19**(9):434-442.

29. Renedo A, Marston C: **Spaces for citizen involvement in healthcare: an ethnographic study**. *Sociology* 2014.

30. Renedo A, Marston C: **Developing patient-centred care: an ethnographic study of patient perceptions and influence on quality improvement**. *BMC Health Services Research* 2015, **15**(22).

31. Renedo A, Marston C: **Healthcare Professionals' Representations of 'Patient and Public Involvement' and Creation of 'Public Participant' Identities: Implications for the Development of Inclusive and Bottom-Up Community Participation Initiatives**. *Journal of Community & Applied Social Psychology* 2011, **21**(3):268-280.

32. Renedo A, Marston CA, Spyridonidis D, Barlow J: **Patient and Public Involvement in Healthcare Quality Improvement: How organizations can help patients and professionals to collaborate**. *Public Management Review* 2015, **17**(1):17-34.

33. Spyridonidis D, Hendy J, Barlow J: **Understanding hybrid roles: the role of identity processes amongst physicians**. *Public Administration Journal* 2014.

34. Caldwell S, Mays N: **Studying policy implementation using a macro, meso and micro frame analysis: the case of the Collaboration for Leadership in Applied Health Research & Care (CLAHRC) programme nationally and in North West London**. *Health Research Policy and Systems* 2012, **10**(32).

35. Spyridonidis D, Hendy J, Barlow J: **Organizational birth: an identity based perspective**. In: *72nd Annual Meeting of the Academy of Management Conference: July 2012 2012; Boston*; 2012.

36. Spyridonidis D, Hendy J, Barlow J: **Organizational identity and ambiguity: A process-based perspective**. In: *26th annual conference for the British Academy of Management: 11-13 September, 2012 2012; Cardiff*; 2012.

37. Spyridonidis D, Hendy J, Barlow J: **Power and Identity in Explaining Transformational Change: An Organizational Analysis**. In: *27th EGOS Colloquium: 2011; Gothenburg, Sweden*; 2011.

38. Pomeroy L: **The Evolution of Knowledge Transfer Boundary Networks in Healthcare**. London: Imperial College London; 2014.

39. Bryant A, Charmaz K: **The Sage handbook of grounded theory**: Sage; 2007.

40. Cooksey SD: **A review of UK health research funding**. In*.* London: Department of Health; 2006.

41. Greenhalgh T, Robert G, Macfarlane F, Bate P, Kyriakidou O: **Diffusion of innovations in service organizations: systematic review and recommendations.** *The Milbank quarterly* 2004, **82**:581-629.

42. Health Do: **Report of the High Level Group on Clinical Effectiveness**. In*.* London; 2007.

43. Langley GJ, Moen R, Nolan KM, Nolan TW, Norman CL, Provost LP: **The improvement guide: a practical approach to enhancing organizational performance**: John Wiley & Sons; 2009.

44. Graham ID, Logan J, Harrison MB, Straus SE, Tetroe J, Caswell W, Robinson N: **Lost in knowledge translation: time for a map?** *Journal of continuing education in the health professions* 2006, **26**(1):13-24.

45. Greenhalgh T, Wieringa S: **Is it time to drop the ‘knowledge translation’metaphor? A critical literature review**. *Journal of the Royal Society of Medicine* 2011, **104**(12):501-509.

46. Nutley S, Walter I, Davies HTO: **From Knowing to Doing: A Framework for Understanding the Evidence-into-Practice Agenda**. *Evaluation* 2003, **9**(2):125-148.

47. Kitson A, G. H, B. M: **Enabling the implementation of evidence based practice: a conceptual framework**. *Quality in health care : QHC* 1998, **7**(3):149--158.

48. Rycroft-Malone J, Harvey G, Kitson A, McCormack B, Seers K, Titchen A: **Getting evidence into practice: ingredients for change**. *Nursing standard (Royal College of Nursing (Great Britain) : 1987)* 2002, **16**(37):38-43.

49. Damschroder LJ, Aron DC, Keith RE, Kirsh SR, Alexander JA, Lowery JC: **Fostering implementation of health services research findings into practice: a consolidated framework for advancing implementation science**. *Implementation Science* 2009, **4**.

50. May C, Finch T: **Implementing, embedding, and integrating practices: an outline of normalization process theory**. *Sociology* 2009, **43**(3):535-554.

51. Batalden PB, Godfrey MM, Nelson EC: **Quality by design: a clinical microsystems approach**. San Francisco: Jossey-Bass; 2007.

52. Nelson EC, Batalden PB, Godfrey MM, Lazar JS: **Value by design: developing clinical microsystems to achieve organizational excellence**: John Wiley & Sons; 2011.

53. Foy R, Graeme M, Jeremy G, Gillian P, Marion C, Richard G: **Attributes of clinical recommendations that influence change in practice following audit and feedback**. *Journal of clinical epidemiology* 2002, **55**(7):717--722.

54. Kaplan HC, P. PL, M. FC, A. MP: **The Model for Understanding Success in Quality (MUSIQ): building a theory of context in healthcare quality improvement.** *BMJ quality \& safety* 2011.

55. Auerbach AD, Landefeld CS, Shojania KG: **The tension between needing to improve care and knowing how to do it**. *New England Journal of Medicine* 2007, **357**(6):608-613.

56. Shojania KG, Grimshaw JM: **Evidence-based quality improvement: the state of the science.** *Health affairs (Project Hope)* 2005, **24**(1):138--150.

57. Perla RJ, Provost LP, Parry GJ: **Seven propositions of the science of improvement: exploring foundations**. *Quality Management in Healthcare* 2013, **22**(3):170-186.

58. Benning a, Ghaleb M, Suokas a, Dixon-Woods M, Dawson J, Barber N, Franklin BD, Girling a, Hemming K, Carmalt M *et al*: **Large scale organisational intervention to improve patient safety in four UK hospitals: mixed method evaluation**. *Bmj* 2011, **342**:d195-d195.

59. Dixon-Woods M, Bosk CL, Aveling EL, Goeschel CA, Pronovost PJ: **Explaining Michigan: Developing an Ex Post Theory of a Quality Improvement Program**. *Milbank Quarterly* 2011, **89**(2):167-205.

60. Walshe K: **Understanding what works--and why--in quality improvement: the need for theory-driven evaluation.** *International Journal for Quality in Health Care* 2007, **19**(2):57--59.

61. Deming WE: **Out of the Crisis**. Cambridge, Massachusetts: The MIT Press; 2000.

62. Plsek PE, Greenhalgh T: **Complexity science: The challenge of complexity in health care**. *BMJ* 2001, **323**:625-628.

63. McDaniel Jr RR, Lanham HJ, Anderson RA: **Implications of complex adaptive systems theory for the design of research on health care organizations**. *Health care management review* 2009, **34**(2):191.

64. Prigogine I: **Order out of chaos: Man's new dialogue with nature**. 1984.

65. von Bertalanffy L: **Perspectives on General Systems Theory**. New York: George Braziller Inc.; 1975.

66. Senge P: **The fifth discipline: The art and practice of the learning organisation.** London: Random House; 2006.

67. Ferlie E, Fitzgerald L, Wood M, Hawkins C: **The nonspread of innovations: The mediating role of professionals**. *Academy of Management Journal* 2005, **48**(1):117-134.

68. Ferlie EB, Shortell SM: **Improving the quality of health care in the United Kingdom and the United States: a framework for change.** *The Milbank quarterly* 2001, **79**:281-315.

69. Waring J, Currie G: **Managing expert knowledge: organizational challenges and managerial futures for the UK medical profession**. *Organization Studies* 2009, **30**(7):755-778.

70. Gagné M, Deci EL: **Self‐determination theory and work motivation**. *Journal of Organizational behavior* 2005, **26**(4):331-362.

71. Bridges W: **Managing transitions: Making the most of change**: Da Capo Press; 2009.

72. Brown JS, Duguid P: **Organizational learning and communities-of-practice: Toward a unified view of working, learning, and innovation**. *Organization Science* 1991, **2**(1):40-57.

73. Ham C, Kipping R, McLeod H: **Redesigning work processes in health care: lessons from the National Health Service**. *The Milbank Quarterly* 2003, **81**(3):415-439.

74. Walshe K: **Reorganisation of the NHS in England**. *British Medical Journal* 2010, **341**.

75. Wachter RM, Pronovost PJ: **Balancing “No Blame” with Accountability in Patient Safety**. *New England Journal of Medicine* 2009, **361**(14):1401-1406.

76. Reason J: **Human error: models and management**. *BMJ* 2000, **320**(7237):768-770.

77. Vincent C, Burnett S, Carthey J: **The measurement and monitoring of safety**. In*.*: The Health Foundation; 2013.

78. Frenk J, Chen L, Bhutta ZA, Cohen J, Crisp N, Evans T, Fineberg H, Garcia P, Ke Y, Kelley P: **Health professionals for a new century: transforming education to strengthen health systems in an interdependent world**. *The lancet* 2010, **376**(9756):1923-1958.

79. Lucas B: **Getting the improvement habit**. *BMJ Qual Saf* 2016, **25**(6):400-403.

80. Gabbay J, Le May A, Connell C, Klein K: **Skilled for Improvement?** London: Health Foundation; 2014.

81. Mezirow J: **Perspective transformation**. *Adult education* 1978, **28**(2):100-110.

82. Michie S, van Stralen MM, West R: **The behaviour change wheel: a new method for characterising and designing behaviour change interventions**. *Implementation Science* 2011, **6**(1):42.

83. Cane J, O’Connor D, Michie S: **Validation of the theoretical domains framework for use in behaviour change and implementation research**. *Implementation science* 2012, **7**(1):37.

84. Bovey WH, Hede A: **Resistance to organisational change: the role of defence mechanisms**. *Journal of Managerial Psychology* 2001, **16**(7):534-548.

85. Provost LP: **Analytical studies: a framework for quality improvement design and analysis**. *BMJ quality & safety* 2011, **20**(Suppl 1):i92-i96.

86. Shewhart WA, Deming WE: **Statistical method from the viewpoint of quality control**: Courier Corporation; 1939.

87. Gibbons M, Limoges C, Nowotny H, Schwartzman S, Scott P, Trow M: **The New Production of Knowledge**. London: Sage; 1994.

88. Dewey J: **The essential Dewey, Pragmatism, education, democracy, vol. I**. In*.*: Indiana University Press; 1998.

89. Hacking I: **Representing and intervening: Introductory topics in the philosophy of natural science**. Cambridge: Cambridge University Press; 1983.

90. Oborn E, Barrett M, Racko G: **Knowledge translation in healthcare: A review of the literature**. *Cambridge, UK* 2010.

91. Ferlie E, Crilly T, Jashapara A, Peckham A: **Knowledge mobilisation in healthcare: a critical review of health sector and generic management literature**. *Social Science & Medicine* 2012, **74**(8):1297-1304.

92. Cohen WM, Levinthal DA: **Absorptive capacity: a new perspective on learning and innovation**. *Administrative science quarterly* 1990:128-152.

93. Glaser B, Strauss A: **Applying grounded theory**. *The discovery of grounded theory: strategies of qualitative research Hawthorne, NY: Aldine Publishing Company* 1967:237-251.

94. Strauss A, Corbin J: **Grounded theory methodology**. *Handbook of qualitative research* 1994, **17**:273-285.

95. Strübing J: **Research as pragmatic problem-solving: The pragmatist roots of empirically-grounded theorizing**. *The Sage handbook of grounded theory* 2007:580-602.

96. Reichertz J: **Abduction: The logic of discovery of grounded theory**. *The SAGE handbook of grounded theory* 2007:214-228.

97. Kelle U: **"Emergence" vs. "Forcing" of Empirical Data? A Crucial Problem of "Grounded Theory" Reconsidered**. *2005* 2005, **6**(2).

98. Langley A: **Strategies for theorizing from process data**. *Academy of Management review* 1999, **24**(4):691-710.

99. Reed J, Green S, Howe C: **Translating evidence in complex systems: A comparative review of implementation and improvement frameworks** *International Journal of Quality in Healthcare* under review.

100. Holton JA: **Grounded theory as a general research methodology**. *The grounded theory review* 2008, **7**(2):67-93.
