# Supplementary material for: Simple rules for evidence translation in complex systems: A qualitative study
Source: BMC Med. 2018 Jun 20;16:92. doi: 10.1186/s12916-018-1076-9 (PMC6009041; doi:10.1186/s12916-018-1076-9)
Supplement: Supplementary file 3 — Example of coding and theory development (DOCX 14 kb) [file 12916_2018_1076_MOESM3_ESM.docx]

S3 File. Example of coding and theory development

| Conceptual Framework Category | |
| --- | --- |
| Strategic principle – Act scientifically and pragmatically | |
| Substantive Theory Category | |
| *Challenge*  It cannot be assumed that interventions will be used or achieve the desired improvement  *Operational principle to guide action*  Test and iteratively develop potential solutions | |
| Categories and relationships identified through axial coding | Examples of themes identified through open coding |
| Challenges   - Multiple reasons affect the uptake of an intervention - Challenges emerge throughout the change process - Practices or processes of care are inter-related - Changes have unintended consequences   Effective strategies for overcoming challenges   - Incremental experimental approach to introduce changes - Use formal and informal methods to study progress and modify improvement approach and refine change theory - Identify adverse effects in other parts of the system | - Unanticipated issues and emerging learning - Identification of broken or problematic processes that prevented intervention uptake, effectiveness etc - Influence of context on intervention uptake and effectiveness (related processes and practices, support from staff and organisation, political, financial and strategic drivers) - Influence of staff and patient perceptions on uptake and use including resistance and promotion - Insights from staff and patients on problems and opportunities for improvement - Assessing and responding to uptake, use, effectiveness and efficiency of intervention including progress against intended and unintended consequences - Creating situations to encourage discussion and learning, identification of unexpected developments and obtaining feedback from staff modifying intervention(s) to fit with processes - Speed at which challenges are identified and responded to - Scale of testing including time, resource, opportunity for learning - Ability to modify intervention in response to learning - Modifying processes to support fit of intervention(s) - Incorporating aspects of established norms, practices, behaviours or preferences into intervention(s) - Modifying current practice and behaviours (e.g. through education, role modelling, peer-to-peer influence) - Modifying intervention(s) characteristics, processes or practices to meet resource or financial constraints - Modifying resource or financial constraints to support effective use of intervention(s) |
